# Stress induced aging in mouse eye

**DOI:** 10.1111/acel.13737

**Published:** 2022-11-17

**Authors:** Qianlan Xu, Cezary Rydz, Viet Anh Nguyen Huu, Lorena Rocha, Claudia Palomino La Torre, Irene Lee, William Cho, Mary Jabari, John Donello, David C. Lyon, Robert T. Brooke, Steve Horvath, Robert N. Weinreb, Won‐Kyu Ju, Andrzej Foik, Dorota Skowronska‐Krawczyk

**Affiliations:** ^1^ Department of Physiology and Biophysics, Center for Translational Vision Research, School of Medicine University of California, Irvine Irvine California USA; ^2^ Viterbi Family Department of Ophthalmology, Hamilton Glaucoma Center and Shiley Eye Institute, School of Medicine University of California, San Diego La Jolla California USA; ^3^ Department of Anatomy and Neurobiology, School of Medicine University of California, Irvine Irvine California USA; ^4^ Epigenetic Clock Development Foundation Torrance California USA; ^5^ International Centre for Translational Eye Research, Institute of Physical Chemistry Polish Academy of Sciences Warsaw Poland; ^6^ Department of Physiology and Biophysics, Department of Ophthalmology, Center for Translational Vision Research, School of Medicine University of California, Irvine California USA

**Keywords:** aging, IOP, retinal ganglion cells, senescence, stress response

## Abstract

Aging, a universal process that affects all cells in an organism, is a major risk factor for a group of neuropathies called glaucoma, where elevated intraocular pressure is one of the known stresses affecting the tissue. Our understanding of molecular impact of aging on response to stress in retina is very limited; therefore, we developed a new mouse model to approach this question experimentally. Here we show that susceptibility to response to stress increases with age and is primed on chromatin level. We demonstrate that ocular hypertension activates a stress response that is similar to natural aging and involves activation of inflammation and senescence. We show that multiple instances of pressure elevation cause aging of young retina as measured on transcriptional and DNA methylation level and are accompanied by local histone modification changes. Our data show that repeated stress accelerates appearance of aging features in tissues and suggest chromatin modifications as the key molecular components of aging. Lastly, our work further emphasizes the importance of early diagnosis and prevention as well as age‐specific management of age‐related diseases, including glaucoma.

## Graphical abstract

Skowronska‐Krawczyk and colleagues describe the transcriptional and epigenetic changes happening in aging retina. They also show that upon stress such as intraocular pressure elevation in the eye, retinal tissue undergoes epigenetic and transcriptional changes similar to natural aging. Finally, they demonstrate that upon repetitive stress young retina shows features of accelerated aging that can be measured using unbiased methods such as DNA methylation clock.

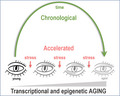

## 1 INTRODUCTION

Aging is a complex process for which distinct molecular aspects contribute to age‐related tissue dysfunction. Systematic analysis of epigenomic and transcriptomic changes across mouse tissues during aging identified several recurring processes including interferon alpha response, IL6‐JAK‐STAT3 signaling, complement, and other components of innate immune response being gradually upregulated with age (Benayoun et al., 2019). These changes are conserved as the molecular signatures of aging and are consistently upregulated in mice and other vertebrates in the absence of pathogens.

Glaucoma, an age‐related eye disease (Weinreb et al., 2014), is characterized by progressive neurodegeneration of the optic nerve that if untreated leads to morphological changes, functional decline, and eventual blindness. Age is the most consistent risk factors for glaucoma. By contrast, other risk factors, including elevated intraocular pressure (IOP), family history, and high myopia, exhibit more variability. Because of the rapid increase in aging populations worldwide, current estimates show that the number of people with glaucoma (aged 40–80) will increase to over 110 million in 2040 (Allison et al., 2020).

Until now, lowering IOP is the only approved and effective treatment paradigm. However, many treated glaucoma patients continue to experience deterioration of vision and progress to blindness, highlighting the critical need to understand its underlying mechanisms (Skowronska‐Krawczyk et al., 2015; Weinreb et al., 2014; Weinreb et al., 2016).

Pathophysiological stress related to elevated IOP induces a broad spectrum of changes in the connective tissues and blood vessels that are central to the function of the retina. They also induce molecular changes in retinal cells, including retinal ganglion cells (RGCs). We have shown previously that with experimental ocular hypertension, RGCs respond to stress by expressing *p16Ink4a*, assume a senescent phenotype, and secrete molecules known as senescence‐associated secretory phenotype (SASP) such as IL6. Similarly, we have observed an increase of senescent cells in our mouse model of ocular hypertension and in human glaucomatous retinas (Skowronska‐Krawczyk et al., 2015). Senescence is a type of damage response that develops in cells experiencing irreparable stress for a sustained period of time, and it is commonly observed in all aged tissues, including the eye. Therefore, we investigated the extent to which aging contributes to IOP‐related senescence induction, stress, and RGC death upon IOP elevation.

Age‐related sterile inflammation, commonly termed “Inflammaging,” has been reproducibly observed in many laboratories. In this work, we describe functional and molecular changes in the aging retina, showing changes characteristic of inflammaging, as found in other tissues (Franceschi et al., 2018), and strong downregulation of retinal lipid metabolism. We show that aged retinas are more sensitive to mild IOP elevation and that old RGCs express significant numbers of senescence markers upon IOP‐associated stress. Next, we demonstrate that aged retinas are prone to respond to the insult more robustly than young tissues on pre‐opened enhancers, and this correlates with differences in chromatin modification upon stress. Finally, we show that multiple mild instances of IOP elevation in young animals preprogram the tissue to respond more strongly to stress, similar to the response of the aged tissue. Our DNA methylation analysis revealed that multiple mild stresses on the young retinas increases DNA methylation age, suggesting the direct influence of repetitive stress on retina aging.

Our results collectively suggest that throughout the life of the animal, retinal cells accumulate the competence on the epigenetic level to respond to stress faster and stronger, eliciting more damage to the tissue in the form of the inflammation and senescence, including the secretion of SASPs and destabilization of the extracellular matrix. These results might explain the difficulties in managing the responses to glaucomatous insults, such as elevated IOP, in aged individuals and suggest novel approaches to manage age‐related eye diseases, including glaucoma.

## 2 RESULTS

### 2.1 Functional and molecular changes in aging retina

First, the impact of aging on visual functions in young (3‐month‐old) and old (18‐month‐old) mice was measured using several functional assays.

**FIGURE 1:**
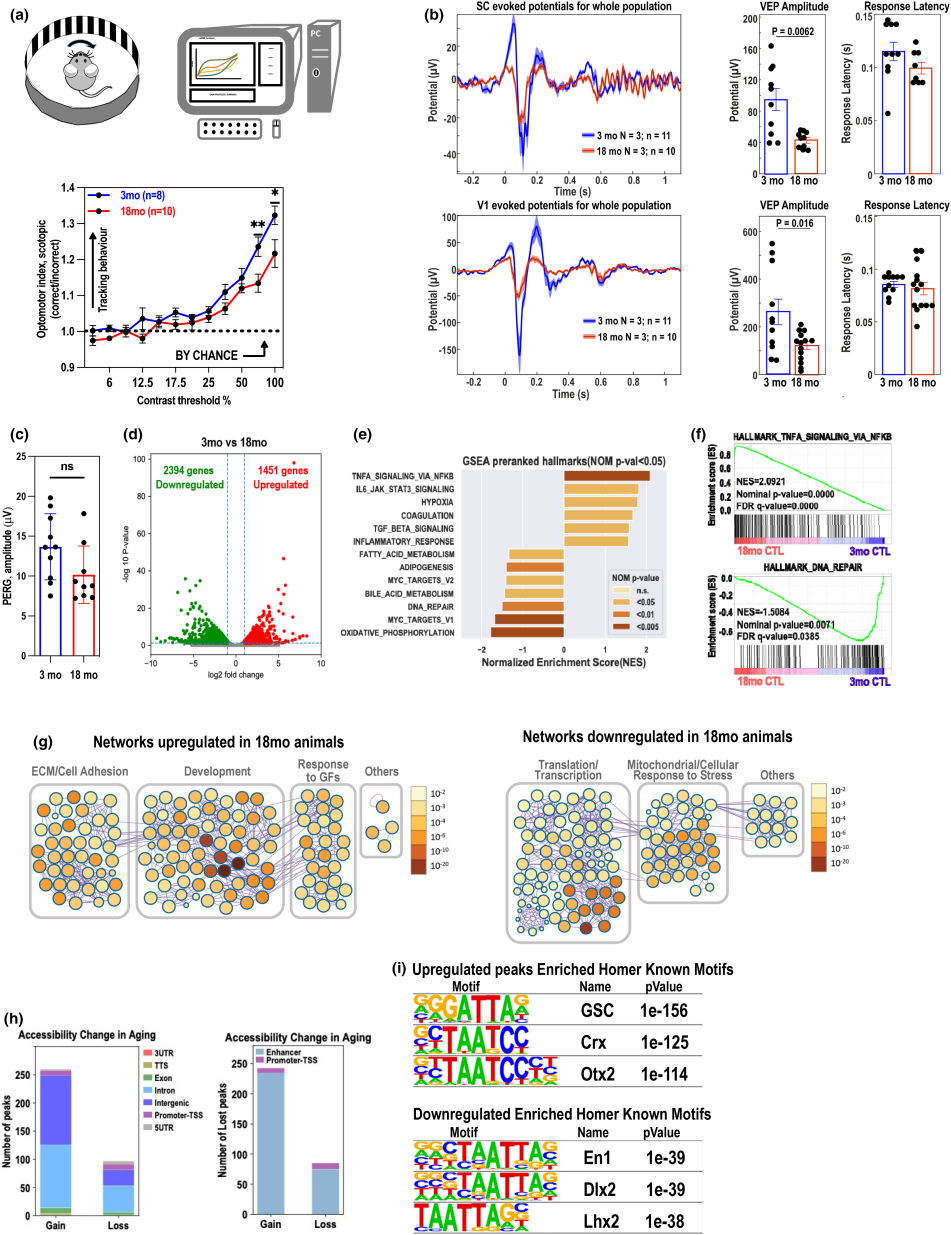
Functional and molecular changes in aging retina. (a) Schematic of the optomotor response (OMR) instrument. (top) OMR at various pattern contrast (bottom). Evaluation of response is automatic and presented as OMR index (ratio of correct/incorrect). Statistically significant tracking obtained using one‐tailed test. 3‐month‐old (*n* = 8) and 18‐month‐old (*n* = 10) animals were analyzed. Mean and SEM. are shown. *p < 0.05, **p < 0.01. (b) Visually evoked potential recordings. VEPs were recorded in superior colliculus (SC) left‐top, and in primary visual cortex (V1) left‐bottom. VEP amplitudes and response latencies (right‐top, right‐bottom) are presented. 3‐month‐old (*n* = 11) and 18‐month‐old (*n* = 10) animals were analyzed. Unpaired *t* test. Mean and SD are shown. (c) PERG performed on 3‐month‐old (*n* = 10 eyes) and 18‐month‐old (*n* = 9 eyes) wild‐type mice shows no significant change in amplitude in aged animals. (unpaired, two‐tailed *t* test, mean and SD are shown). (d) Volcano plots of differentially expressed genes between 3‐month‐old and 18‐month‐old non‐treated wildtype mouse retinae. Each dot represents one gene with −log10(adjusted *p*‐value) versus log2foldchage. Dots in grey represent no significant genes while the dots highlighted in red and green respectively represent up‐regulated and down‐regulated DEGs. *n* = 2. (e) The bar plot showed normalized enrichment scores (NES) of all defined significant enriched (nominal *p*‐value <5%) hallmark gene sets from pre‐ranked GSEA in 18‐month‐old non‐treated wildtype mouse retinae compared to the 3‐month‐old retinae. *n* = 2 biological replicates. NES determines the magnitude of enrichment of each hallmark gene set across all analyzed gene sets. The statistical significance in nominal (NOM) *p*‐value was indicated by a discrete color scale. (f) GSEA enrichment plots of selected top‐ranked hallmark pathways. (g) Networks of enriched pathway from up‐ and down‐regulated DEGs during aging using Metascape. Each node in the figure represents one enriched ontology term. Node size indicates the number of genes in each term. The statistical significance in FDR q‐value was shown by a discrete color scale for each node. All the enriched terms were further bounded and annotated as more general classes. (h) Stacked bar plots showed up‐ and down‐regulated accessible regions with cutoffs at 0.05 in FDR and two‐fold change of normalized ATAC‐seq signal. Specific genomic regions were color coded. (i) Motif analyses using tool Homer identified potential regulators during retina aging. Regions of accessibility gain or loss were enriched in Homer known motifs. The table showed top‐ranked potential transcription factors in significance by motif analysis of ATAC‐seq data in comparison between 3‐month‐old and 18‐month‐old mouse retinae.

The quantitative optomotor response (OMR) analysis was performed to measure contrast sensitivity in scotopic (nighttime) and photopic (daytime) light level conditions. In this assay, a mouse is placed on a platform where it can move and track a stimulus—rotating pattern displayed on a screen (Figure 1a, top). Response evaluation is automatic and presented as an OMR index (ratio of correct/incorrect). Contrast sensitivity was defined as the inverse of contrast threshold for OMR, and measurements were performed using an automated, commercially available setup (Figure 1a). Our data show that old animals have significantly lower contrast sensitivity in both scotopic and photopic conditions relative to young animals (Figure 1a, Figure S1A). For example, in low luminance of ~0.03 lux (nighttime light levels) the tracking behavior of aged animals was decreased already at 100% contrast. In daytime light levels, 18‐month‐old healthy animals exhibited significantly decreased tracking response already at 50% contrast when compared to the younger animals (Figure S1A).

Next, we evaluated age‐related effects on the integrity of the visual pathway by recording visually evoked potentials (VEP) in young and old animals. The recordings were performed in the superior colliculus (SC) and the primary visual cortex (V1). In both regions, there was a significant reduction in VEP in 18‐month‐old mice (red trace) compared to the 3‐month‐old (blue trace) animals. For example, in the SC, the VEP amplitude dropped by 56% from 95 ± 14 μV in 3‐month to 42 ± 4 μV in 18‐month‐old animals (*p* = 0.006). In V1, the drop was 60%, from 263 ± 54 μV in 3‐month to 106 ± 12 μV in 18‐month‐old animals (*p* = 0.016). On the other hand, response latencies were comparable between age groups in both regions (115 ± 7 ms vs. 99 ± 6 ms in the SC; and 85 ± 3 ms vs.81 ± 6 ms in the V1; Figure 1b).

To examine the extent to which the observed decrease of visual function can be attributed to the loss of RGC activity, the pattern electroretinography (PERG) responses were measured. PERG signals (OS) from ten 3‐month and nine 18‐month‐old animals were recorded. Our findings demonstrate no significant difference in PERG amplitudes between young (mean, 13.67 ± 4.14 μV [SD]; *n* = 10 eyes) and old animals (mean, 10.17 ± 3.59 μV; *n* = 9 eyes; *t* = 1.956, *p* = 0.0671; Figure 1c).

To investigate whether the molecular changes in the aging retina can explain the observed functional decline of the visual system, we performed a whole retina mRNA sequencing on retinas isolated from both 3‐month and 18‐month‐old mice. We found that there were 1451 genes significantly up‐regulated (log2 fold ≥1, FDR ≤0.05) and 2394 genes significantly down‐regulated (log2 fold ≤−1, FDR ≤0.05) upon natural aging (Figure 1d). The pre‐ranked Gene Set Enrichment Analysis (GSEA) revealed a dysregulation in several conserved pathways (MsigDB hallmark collection version v7.5.1) in aged animals (Liberzon et al., 2015). In particular, we found a significant upregulation of inflammatory response (Normalized enrichment score [NES] = 1.56), NF‐kB regulated TNF alpha signaling (NES = 2.09), and IL‐6 receptor signaling (NES = 1.82) and a significant downregulation of DNA repair (NES = −1.51), fatty acid metabolism (NES = −1.34), and oxidative phosphorylation pathways (NES = −1.78) in the 18‐month‐old mice relative to the 3‐month‐old animals (Figure 1e,f).

Metascape enrichment network (Zhou et al., 2019) visualized the intra‐ and inter‐relationships among the up‐ and down‐regulated gene pathways in natural aging (3‐month‐old versus 18‐month‐old; Figure 1g, Figure S1B). capturing the clustered upregulation of processes such as extracellular matrix organization, response to growth factors, and general development. Downregulated clusters included stress‐related mitochondria organization and translation.

To compare transcriptomic data with epigenetic changes during natural aging, we performed an Assay for Transposase‐Accessible Chromatin with high‐throughput sequencing (ATAC‐Seq) analysis on 3‐month‐old (*N* = 6) and 18‐month‐old (*N* = 6) mouse retinas. The epigenetically affected genes were determined by the significant differential chromatin accessibility in both promoter‐TSS and the nearest enhancer regions (Figure S1C).

The open chromatin regions or significant reads coverage (*q*‐value ≤0.05) of ATAC‐Seq data were identified by peak calling using MACS2 (Model‐based Analysis for ChIP‐Seq, version 2.2.7.1). The chromatin accessibility of the young and old retina samples was compared by using differential analysis tool edgeR in the count matrix built from the consensus list of detected open regions from all samples with significance cutoff at 0.05 in FDR q‐value. 260 regions gained accessibility uniquely in 18‐month‐old retina (total 394) while only 15 regions gained accessibility uniquely in 3‐month‐old mouse retina (total 121) showing significant overlap in open sites upon IOP between stages (Figure 1h). Majority of the age‐related epigenetically remodeled regions were not on the promoters but on enhancer regions. Similarly, loss of the accessible regions only partially (~10%) overlapped with promoters of the genes. Transcription factor (TF) motif analysis indicated enrichment of unique sets of homeodomain transcription factors in both groups (Figure 1I, Figure S1D). In particular, predicted binding sites for Crx and Otx2, TFs involved in development and maintenance of the photoreceptor cells are highly enriched.

### 2.2 Aged retina is more sensitive to mild intraocular pressure elevation

In our previous works, we have demonstrated that intraocular hypertension of 90 mmHg, non‐physiological levels of pressure, induced ~50% loss of RGCs (Skowronska‐Krawczyk et al., 2015). Therefore, in search of a better system that would mimic the glaucomatous insult, the effects of different levels of IOP elevation on RGC survival were quantified, by unilaterally elevating IOP in 3‐month‐old C57BL/6J to 30, 50, or 90 mmHg for 1 h. The contralateral eye served as a healthy sham control. At day 7 post‐IOP, retinas were dissected, fixed, and stained using the antibody against Brn3a or RBPMS, specific markers for RGCs. In comparison with control, we found that only IOP elevated to 50 and 90 mmHg pressure induced a significant decrease in the RGC count. In addition, there was a direct correlation between the magnitude of RGC loss with the level of applied IOP. Mild IOP elevation of 30 mmHg had no impact on RGC count in 3‐month‐old animal after 7 days. To explore whether simple needle puncture can contribute to RGC loss, 7 mm Hg and 15 mmHg pressure cohorts were tested. At 7 days post‐procedure, analysis of the immunostained retinal flat mounts revealed no decrease in RGC count in procedural eyes as compared to healthy contralateral eyes (Figure S2A,B). Importantly, RGC quantification at 21 days post‐IOP elevation depicted similar numbers of preserved RGCs to those of 7 days post‐IOP (Figure S2C,D).

To evaluate the effect of increasing IOP levels on the integrity of the visual pathway, VEP was measured in each group of animals (Figure S2E) and response amplitudes were quantified from the peak‐to‐peak analysis of the first negative component N1 (Figure S2F). Interestingly, there was a decrease in visual potential amplitude compared to contralateral healthy eye in all experimental groups at day 7 post‐IOP. The eyes exposed to 30, 50, and 90 mmHg resulted in reduction of the P1‐N1 but the reduction was significant only for IOP >30 mmHg. In addition, the magnitude of reduction correlated with the level of applied IOP. VEP signals were not affected by the procedure (Figure S2F), and VEP amplitudes in IOP‐treated eyes 21 days post‐IOP were reduced only in groups with IOP elevation >30 mmHg (Figure S2G).

To assess potential effects of the IOP elevation on the temporal properties of VEP response, we measured the change in P1 and N1 time‐to‐peak of the VEP signal. Only the latencies for the 90‐mmHg experimental group showed a significant increase for P1 and N1 component, respectively, at 7 days post‐procedure. Interestingly, this change reverted by day 21 post‐IOP treatment. None of the other groups exhibited P1 or N1 latency change 7 or 21 days post‐IOP (Figure S2H,I).

**FIGURE 2:**
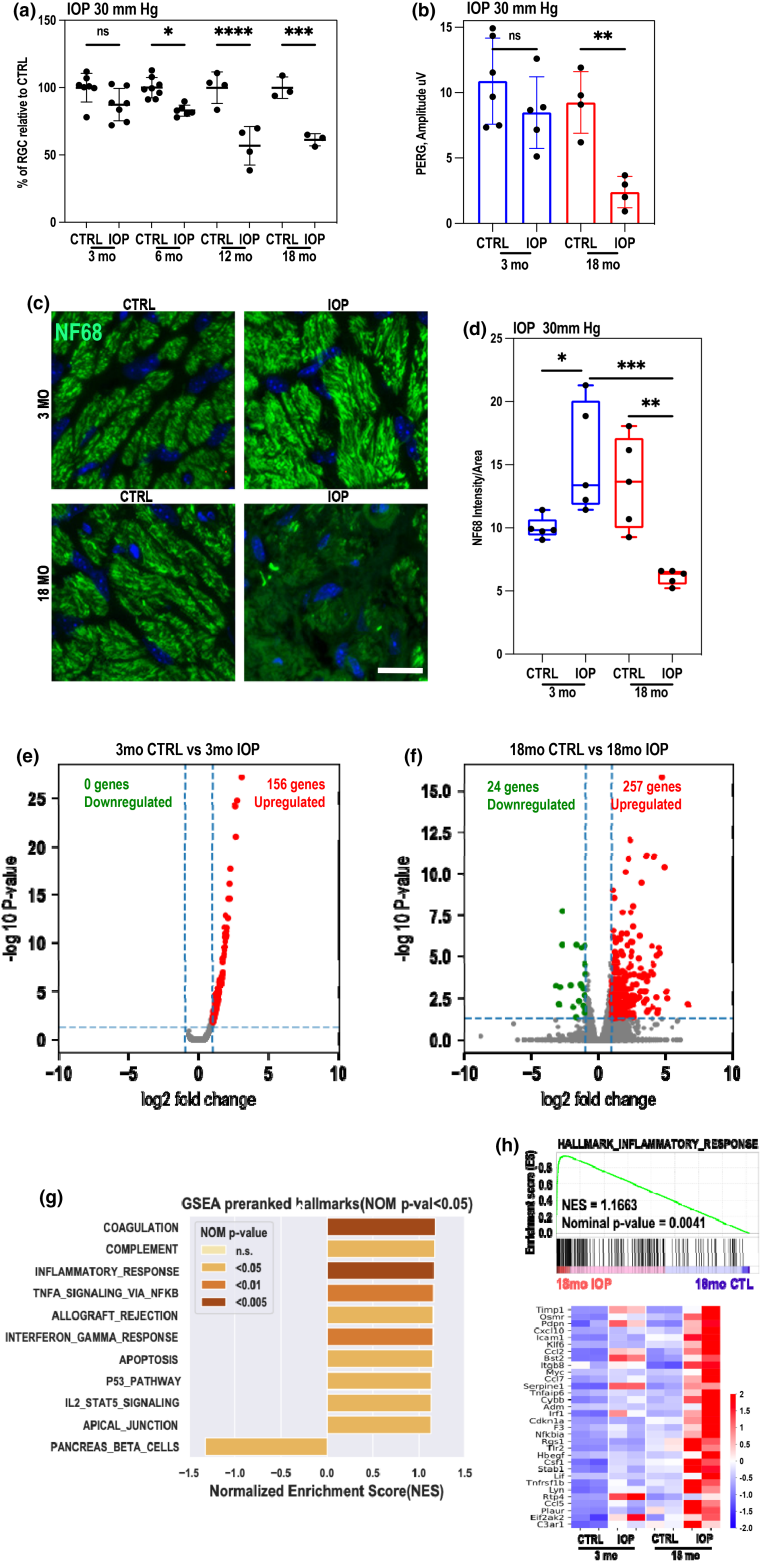
Aged retina is more sensitive to the mild intraocular pressure elevation. (a) Loss of RGC upon stress is more severe in aged animals. Quantification (Brn3a immunostaining) of RGC loss in 3‐month (*n* = 8), 6‐month (*n* = 8), 12‐month (*n* = 4), and 18‐month‐old (*n* = 5) wildtype retinas upon 30 mmHg IOP on day 5 after treatment (one‐way ANOVA, multiple comparisons, mean and SD are shown. *p < 0.05; ***p < 0.001; ****p < 0.0001). (b) PERG performed on IOP‐treated and non‐treated (CTRL) eyes on day 5 after IOP treatment in 3‐month and 18‐month‐old C57BL/6J mice (one‐way ANOVA, multiple comparisons, *p < 0.05; **p < 0.01; ***p < 0.001). (c) NF68 immunohistochemistry in the glial lamina. (top‐left) young mice with non‐IOP elevation. (top right) young mice with IOP elevation. (bottom‐left), aged mice with non‐IOP elevation. (bottom right) aged mice with IOP elevation. (d) Quantitative analyses of NF68 immunoreactivity (*n* = 5 images/group). Mean and SD are shown. Statistical significance determined using one‐way ANOVA. *p < 0.05; **p < 0.01; ***p < 0.001. Scale bar: 100um (all panels). (e, f) Volcano plots of differentially expressed genes between retinas in 3‐month‐old (e) and 18‐month‐old (f) upon 30 mmHg IOP. *n* = 2 biological replicates. The numbers of DEGs with defined significance cut‐offs were marked in the upper space of the plots. (g) Gene set enrichment analysis of RNA‐seq data was performed using GSEA‐Preranked module. *n* = 2 biological replicates. Using the MSigDB hallmark gene set collection (v7.5.1). The bar plot in figure 2g showed all defined significant enriched (nominal *p*‐value <5%) hallmark gene sets from pre‐ranked GSEA in IOP‐treated retina compared to the contralateral non‐treatment retina from the RNA‐seq data were described above. NOM p‐value was shown by a discrete color scale (see scale legend in the figure). (h) Heatmap of row z‐score of RPKM of each gene in gene set: Hallmark inflammatory response (with its pre‐ranked GSEA enrichment plot on top of the heatmap). *N* = 2 biological replicates. The row z‐scores were indicated by a continuous color scale (see scale legend in the figure).

Next, to explore the extent to which the age of the animal influences the impact of IOP elevation on retinal morphology and function, IOP was unilaterally elevated to 30 mmHg for 1 hour in 3‐, 6‐, 12‐, and 18‐month‐old wildtype C57BL/6 mice. At day 7 after IOP elevation, animals were sacrificed, and immunohistochemistry was performed using anti‐Brn3a antibody on flat‐mounted retinas. We observed that in 6‐month, 12‐month, and 18‐month‐old animals, RGC count was significantly lower in the retinas exposed to elevated IOP compared to control retinas. The loss of RGCs was drastically higher in 12‐ and 18‐month‐old animals compared to 3‐month‐old animals, suggesting that RGCs in aged animals are more vulnerable to IOP elevation. (Figure 2a).

To investigate the extent to which age contributes to the loss of RGC function in vision after IOP elevation, IOP was unilaterally raised in 3‐ and 18‐month‐old animals and pattern electroretinogram (pERG), measurement that can capture specific response from RGCs, was performed. At 5 days following IOP elevation, not only was the number of RGCs lower in the treated eyes but also 18‐month‐old animals showed a dramatically reduced PERG amplitude after IOP (mean, 2.39 ± 1.19 μV [SD]; *n* = 4 eyes) in comparison to the control eye (mean, 9.25 ± 2.36 μV; *n* = 4 eyes), whereas in young animals no significant difference between treated and non‐treated eyes was observed (mean, 8.49 ± 2.74 μV; *n* = 5 eyes and mean, 10.89 ± 3.29 μV; *n* = 6 eyes, respectively; Figure 2b).

To further investigate the age‐related impact on visual path, we performed immunohistochemistry in the glial lamina cross‐sections from 3‐ and 18‐month‐old mice treated with IOP elevation (30 mmH) using the antibody for neurofilament 68 (NF68), a marker for axons (Figure 2c). The corresponding areas are marked by dashed line (Figure S2J). In comparison with control young mice with non‐IOP elevation, we first found that elevated IOP increased NF68 immunoreactivity in the glial lamina, showing evidence of insulted RGC axons due to IOP elevation. However, in samples isolated from the treated eyeballs from 18‐month‐old animals, elevated IOP exacerbated axonal degeneration, as observed by a significant reduction of NF68 immunoreactivity in the glial lamina, an effect observed reproducibly in every optic nerve head isolated from the elevated IOP‐treated eye (Figure 2d and Figure S2J). The average region of the axon loss after unique IOP elevation event was assessed to be ~15% and localized in one sector of the optic nerve (Figure S2J *right*).

To capture age‐dependent transcriptional changes in the retinas upon unilateral IOP elevation, RNA‐seq analysis on bulk retinas collected 2 days after the procedure was performed, when cell loss is not yet observed (Rocha et al., 2020). The transcriptome changes were identified using Bioconductor package DESeq2 (Love et al., 2014). We found that in young retinas, only 156 genes were significantly up‐regulated (log2 fold ≥1, FDR ≤0.05), and no genes were significantly down‐regulated upon IOP stress (log2 fold ≤−1, FDR ≤0.05; Figure 2e). In contrast, in aged retinas, 257 genes were significantly up‐regulated (log2 fold ≥1, FDR ≤0.05) and 24 genes were significantly down‐regulated upon 30 mm Hg IOP stress (log2 fold ≤−1, FDR ≤0.05; Figure 2f).

The pre‐ranked GSEA showed no significantly enriched pathways in 3‐month‐old retinas pairs while 9 up‐regulated gene sets and 1 down‐regulated gene set were significantly enriched at nominal *p*‐value <5% between control and IOP treatment in 18‐month‐old retinas (MsigDB hallmark collection version v7.5.1; Figure 2g). These gene sets included a series of inflammatory pathways (TNFα signaling via NFκB, INFγ response, complement, IL2‐STAT5 signaling, and others), p53 and apoptosis pathways and downregulated set of genes recognized as connected with pancreas beta cells pathway. Interestingly, the core genes within significantly enriched pathways (e.g., inflammatory and apoptosis) were robustly upregulated in 18‐month‐old animals upon stress whereas only slightly upregulated in young retinas (Figure 2h, Figure S2K). Similarly, when we plotted heatmaps for senescence and extracellular matrix deregulation pathways (Rocha et al., 2020) we have observed robust upregulation of the genes in these pathways in 18‐month‐old IOP‐treated retinas but only limited deregulation in 3‐month‐old‐treated retinas. In total, out of 65 top genes dysregulated upon IOP elevation in 18‐month‐old retinas, 53 were upregulated more in the old retinas than in the young retinas upon IOP stress (Figure S2L).

**FIGURE 3:**
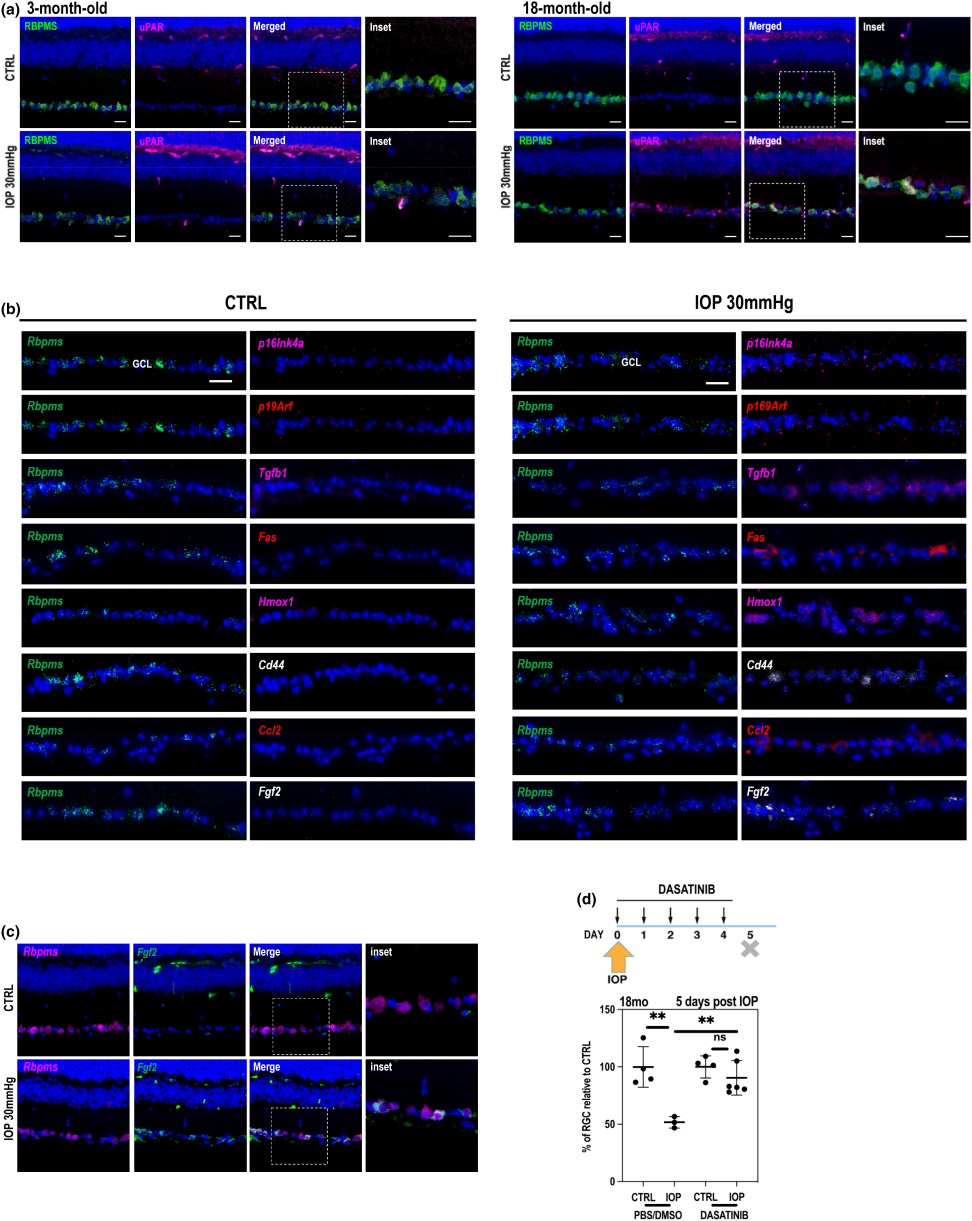
(a) uPAR immunostaining of retinal sections in 3‐month‐old (*left*) and old (*right*) control non‐treated retinas and IOP‐treated retinas. Size bar 20 μm. (b) RNAscope and HiPlex analysis of expression of a set of genes in RGC layer in control non‐treated and IOP‐treated retina. Size bar 20 μm. (c) Immunofluorescence analysis of Fgf2 in old non‐treated retinas and IOP‐treated. Size bar 20 μm. (d) RGC quantification after 5 days of Dasatinib treatment. Statistical significance determined using one‐way ANOVA. **p < 0.01.

Next, we asked whether the dysregulation of expression of genes involved in inflammaging, and senescence detected in bulk RNAseq can be observed in RGCs. For that, 2 days after unilateral 30 mmHg IOP elevation, eyeballs were extracted, fixed and cryosections were collected. First, we performed immunofluorescent staining of young and old retinas using antibody against uPAR, common receptor expressed on senescent cells (Coppé et al., 2008). Our data show that uPAR is specifically expressed in RGCs (Figure 3a), further confirming senescence in these non‐dividing neurons. Next, RNA scope experiment using specific probes for *p16Ink4a*, and *p19Arf* cell cycle inhibitors were used. We have observed upregulation of expression of both genes in retinal ganglion cell layer in retinas isolated from IOP‐treated eyes (Figure S3). Surprisingly, both transcripts were also observed in inner nuclear layer in retinas from stressed eyes. Lastly, we have performed HiPlex assay to detect several transcripts of other genes upregulated in 18‐month‐old retinas upon IOP elevation. We detected all transcripts being upregulated in RGCs (Figure 3b); however, several of them were also upregulated in other retinal cell types (Figure S3). Additionally, immunostaining using anti‐Fgf2 antibody, unequivocally, shows the specific presence of Fgf2 in RGC layer (Figure 3c).

Since we detected increased senescence markers in the 18‐month‐old retina, and especially in RGCs, upon IOP elevation, we decided to test whether we can use a senolytic approach to remove early senescent cells and potentially protect retinal cells from death, as we did previously in young animal upon 90 mmHg IOP (Rocha et al., 2020). Accordingly, we followed a previously established protocol (Figure 3d, *top*), and after unilateral 30 mmHg IOP treatment of the retina, 18‐month‐old animals were intraperitoneally injected daily with a senolytic drug, dasatinib. After 5 days, retinas were collected and immunohistochemistry using anti‐Brn3a antibody was performed on IOP‐treated and non‐treated flat‐mounted retinas. Our quantified data showed a protective effect of a senolytic drug on RGC number after stress caused by IOP elevation (Figure 3d, *bottom*).

### 2.3 Age‐related epigenetic remodeling upon stress facilitates robust transcriptional response

**FIGURE 4:**
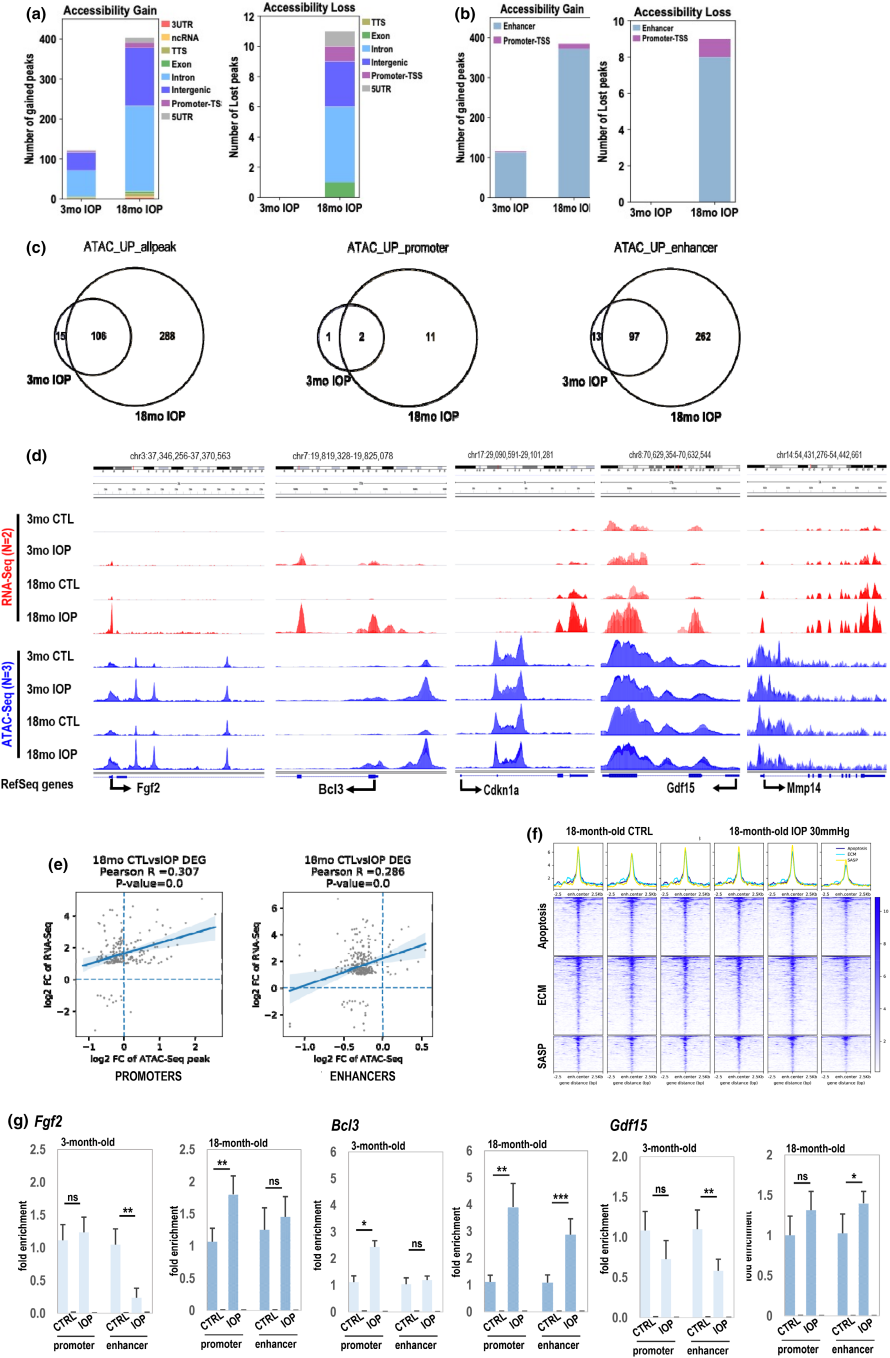
ATAC‐seq analysis of stress‐related epigenetic change. (a, b) stacked bar plots showed gained or lost accessible regions in both comprehensive distribution (a) and cis‐element distribution (b) with cutoffs at 0.05 in FDR and two‐fold change of normalized ATAC‐seq signal. Specific genomic regions were color coded. (c) Venn diagrams showed overlaps of accessibility gain genome‐widely or at enhancers/promoters upon mild IOP stress between 3‐month‐old and 18‐month‐old retinae. (d) The IGV tracks illustrate multiple top‐ranked IOP‐related DE gene locations in 18‐month‐old retinae with dynamic changes in chromatin accessibility by dual factors: Aging and mild IOP. IGV, integrative genomics viewer. (e) R‐square plots illustrate the correlation between transcription activation and chromatin accessibility changes upon mild IOP treatment in 18‐month‐old mouse retinae on promoter‐TSS regions (left) or activity‐by‐contact (ABC) index of both promoters and predicted enhancers with weighted calculation by known *cis*‐looping contacts (right). (f) Profile plots and heatmap plots for chromatin accessibility intensities (deepTools normalized RPKM with bin size 5) over the nearest enhancer regions (2.5 kb flanking distance to center of each region) of curated gene set. In the heatmap plot, higher accessible chromatin regions were shown in darker color (see color legend) and the genomic regions were sorted based on descending order of mean signal value per region in IOP‐treated 18‐month‐old mouse retina. *n* = 3 biological replicates. (g) H3K27Ac ChIP‐qPCR analysis of selected regulatory elements. Statistical significance determined using unpaired *t* test. *N* = 3 biological replicates *p < 0.05; **p < 0.01; ***p < 0.001

To understand the chromatin remodeling events that occur after IOP elevation in young and aged retinas, we performed ATAC‐seq on retinas isolated three days after the 30 mmHg IOP treatment. Similar to transcriptional age‐related differences, we observed more changes in chromatin accessibility upon IOP stress in aged retinas (Figure 4a and Figure S4A). In particular, when we compared the regions specifically open upon stress, almost four times more regions became opened upon stress in chromatin isolated from 18‐month‐old retinas than 3‐month‐old retinas. In both cases, most of the regions were either intronic or intergenic (Figure 4a,b). All of these newly opened regions, after cross‐referencing with mouse genome database, were located in enhancers. Only very few regions, also localized in enhancers, lost accessibility.

Next, we used HOMER analysis (Boeva, 2016; Shiraishi‐Yamaguchi & Furuichi, 2007) to detect predicted TF binding sites in regions specifically open upon IOP elevation in aged animals (Figure S4A). We detected that the top 5 results belonged to STAT family of TFs well known effectors of response to interferons and inflammation. Interestingly, other TF bindings sites significantly enriched on regulatory elements open upon mild IOP in 18mo retinas were those of homeodomain TFs previously detected as enriched on open elements in aged retina when compared to open elements in young retina (Crx and Otx2), (Figure S4B). This finding again shows similarities in aging and stress response program.

Then, we looked at the overlap between the epigenetic programs upon stress in 3‐month‐old and 18‐month‐old retinas (Figure 4c). Immediately, it became apparent that DNA accessibility in young animals almost fully overlaps with that of old animals. We also observed that most stress‐induced changes on the chromatin level occurred on enhancers in both 3‐month‐old and 18‐month‐old animals, with only 262 additional open enhancers found in aged animal.

When we looked at specific examples, we noted that although, some genes highly upregulated specifically in aged retina, only few of them had some changed accessibility in aged retina upon IOP (*Fgf2*, *Bcl3*), while other genes (*Cdkn1a*, *Gdf15*, *Mmp14*) had no change in accessibility in their locus despite significant transcription upregulation (Figure 4d). Then, statistical scoring based on estimating enhancer activity and enhancer‐promoter contact frequency by Activity‐by‐contact (ABC) model was performed on ATAC‐Seq data which was projected on published Hi‐C data in adult mouse retinas (Norrie et al., 2019; Figure 4e). Each dot in R‐square plot represents one activated DE gene with log2foldchange in transcription level versus log2foldchage of accessibility gain at promoter‐TSS region (±2.5 kb; left) or ABC score in the 1 Mb flanking range of promoter‐TSS (right). We found that there was only a mild correlation (*R* = 0.307) between the presence of open enhancers and gene upregulation, similarly mild correlation (*R* = 286) could be detected on promoters (Figure 4e). Interestingly, when we analyzed pathways of interests, such as SASP, ECM deregulation, and apoptosis we noted no change in enhancer openness for genes in these pathways. In fact, these enhancers were already open in non‐treated retina (Figure 4f, Figure S4C). This intriguing data suggest that other level of regulation (DNA modification, histone modification, or other) is involved in final activation of these genes upon signal. We therefore tested histone modification, H3K27Ac, as highly correlated with active regulatory DNA elements. Using chromatin immunoprecipitation followed by qPCR (ChIP‐qPCR) method, we looked at levels of H3K27Ac histone mark at promoters and enhancers of several genes differentially regulated in 18‐ versus 3‐month‐old animals at day 2 after IOP. Our data showed that in old animals, H3K27Ac levels are increased on all tested elements upon stress. In contrast, in young animals only one gene promoter (*Bcl3*) has significantly higher level of positive histone mark, while other tested regulatory elements show lower levels of H3K27Ac (Figure 4g).

### 2.4 Multiple low‐level stress occurrences amplify transcriptional response to mild stress and accelerate DNA methylation aging clock

**FIGURE 5:**
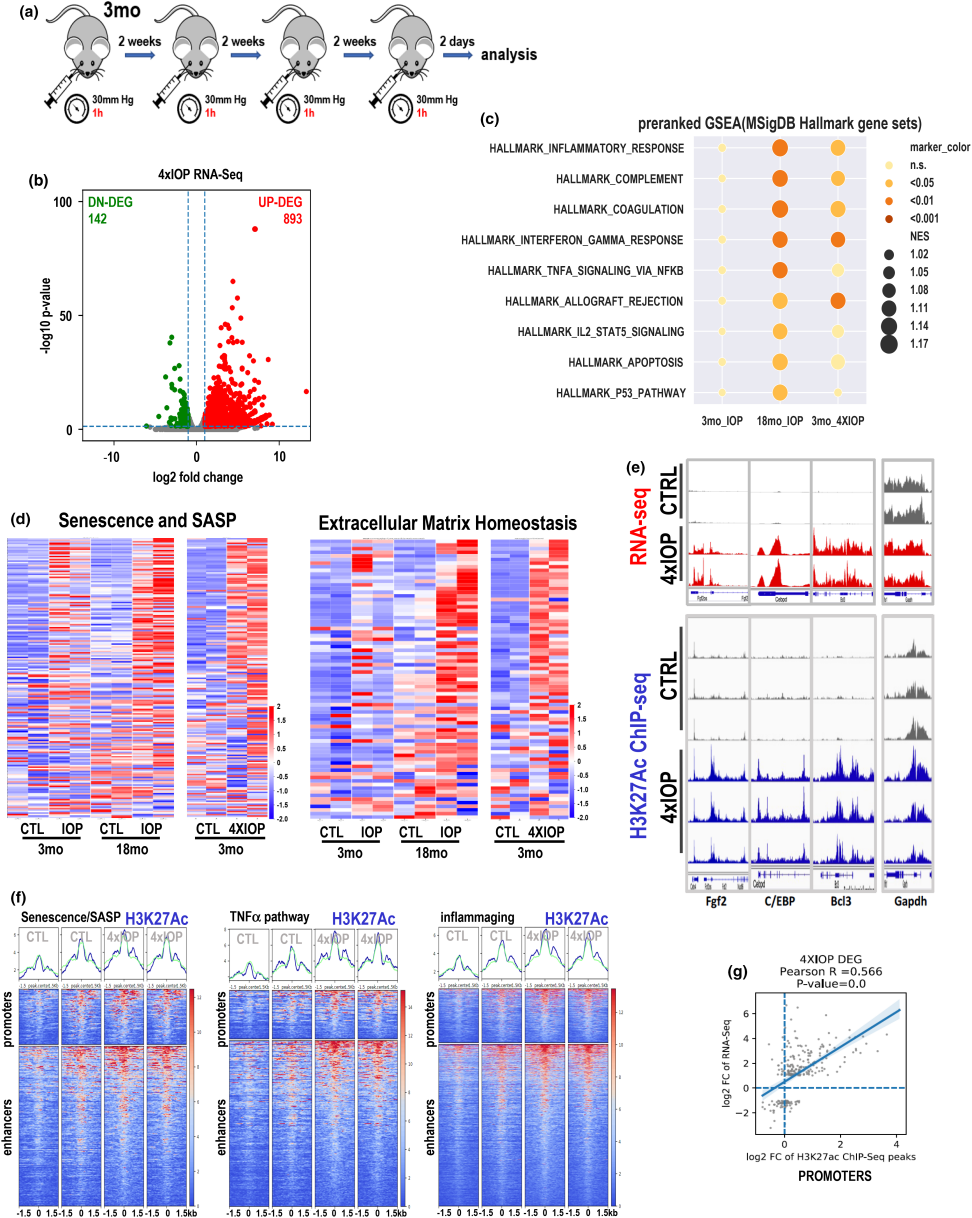
Recurring stress accelerates aging of the retina. (a) Scheme of the experiment. 3‐month‐old mice were exposed to unilateral IOP elevation (30 mmHg) for 1 h. The experiment was repeated every two weeks over 2‐month period, in total 4 unilateral (OD) IOP elevation experiments per animal. The contralateral eye served as a control. Two days after the final IOP, retinas were collected and bulk RNA‐seq was performed. (b) Volcano plots showed the transcriptome change upon multiple times of IOP treatment. *n* = 2 biological replicates. (c) Dot plot shows gene expression regulation pattern by preranked GSEA enrichment analysis of one‐time IOP treatment on 3‐month and 18‐month retinae and multiple IOP treatment on 3‐month retinae. *n* = 2 biological replicates. The NOM *p*‐value of each term is shown by a discrete color scale of the dot. Normalized enrichment score (NES) is shown by dot size (see figure legend). (d) Heatmaps of row z‐score of RPKM of each gene in gene sets: Senescence and SASP and ECM homeostasis with its pre‐ranked GSEA enrichment plot on top of the heatmap and curated senescence pathway genes (right). (e) The IGV tracks illustrate several top‐ranked IOP‐related DE gene locations in 3‐month‐old retinae with dynamic changes in chromatin modifications by dual factors: Aging and 4xIOP. (f) Profile plots and heatmap plots for H3K27Ac enrichment (deepTools normalized RPKM with bin size 5) over the nearest enhancer regions (1.5 kb flanking distance to center of each region) of curated gene set. In the heatmap plot, higher accessible chromatin regions were shown in red (see color legend) and the genomic regions were sorted based on descending order of mean signal value per region in 4xIOP‐treated 3‐month‐old mouse retina. *n* = 2 biological replicates. (g) *R*‐square plot illustrates the high correlation between transcription activation and H3K27Ac enrichment upon repetitive IOP treatment in 3‐month‐old mouse retinae on promoter‐TSS regions.

Next, we investigated whether repeated mild IOP elevation in young animal can affect the expression levels of genes involved in the response to stress. In brief, 3‐month‐old animals were treated with unilateral 30 mmHg IOP elevation for 1 h, four times, every two weeks (Figure 5a). The contralateral eye served as a control. Two days after the final IOP insult, retinas were collected and bulk RNA‐seq was performed. Data analysis showed 893 genes significantly up‐regulated (log2 fold ≥1, FDR <0.05) and 142 genes significantly down‐regulated (log2 fold ≤−1, FDR <0.05) upon repeated stress, in comparison to non‐treated, contralateral eyes (Figure 5b). The pre‐ranked GSEA revealed several pathways involved in inflammatory response, apoptosis, and DNA repair to be significantly upregulated. Interestingly, the list of significantly upregulated pathways closely resembled that of pathways upregulated in 18‐month‐old retinas upon 30 mmHg IOP elevation (Figure 5c, Figure S5A,B). Moreover, when we compared pathways such as senescence and SASPs, ECM deregulation, and apoptosis, the pattern of gene expression in 4xIOP retinas resembled the expression pattern of aged retina after IOP and not that of young retina after the stress (Figure 5d, Figure S5C). This suggests that recurrent stress on young tissue can induce changes in its ability to respond to stress making it transcriptionally similar to aged retina. We therefore hypothesized that dynamics of H3K27Ac levels in young retinas upon repetitive stress will be similar to aged retinas upon one‐time stress. To test this hypothesis, we performed ChIP followed by next‐generation sequencing, ChIP‐seq, using anti‐H3K27Ac antibody. First, we looked at the selected examples and we noted that high levels of this positive mark can be detected at the regulatory regions of genes highly upregulated only upon 4xIOP (Figure 5e). We then looked genome‐wide and prepared heatmaps of regulatory regions of genes included in selected pathways upregulated in retinas upon repetitive stress (Figure S5A). In contrast to ATAC‐seq data in aged retinas upon 1xIOP, H3K27Ac peaks were highly upregulated upon repetitive stress in young retinas in all upregulated genes (Figure S5D) and in selected pathways, including TNFα pathway, inflammaging, and merged senescence/SASP (Figure 5f). In fact, we detected very high correlation of H3K27Ac mark present on promoter and gene activity in retina that underwent several rounds of stress (Figure 5g).

### 2.5 Epigenetic clock analysis

**FIGURE 6:**
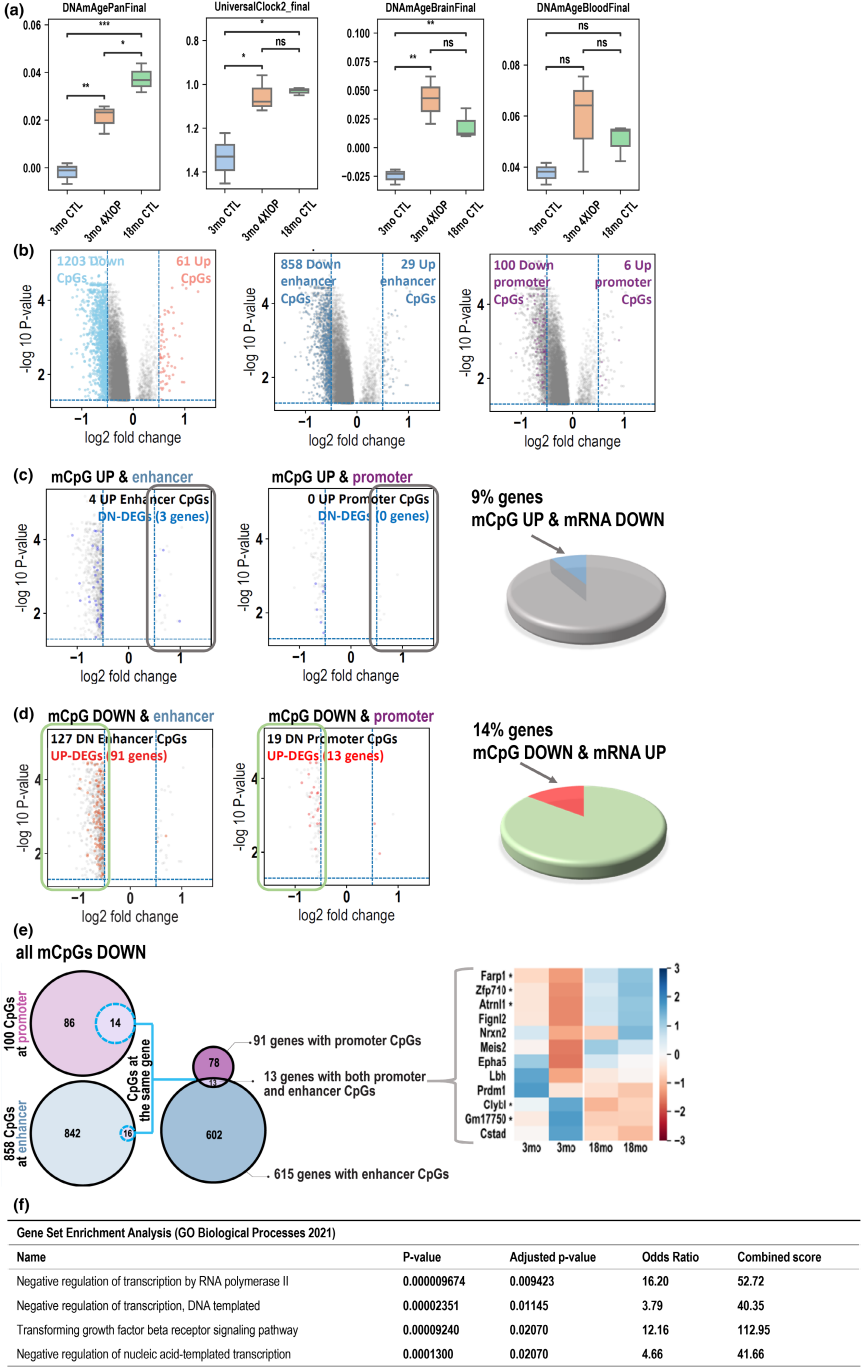
(a) Predicted age (by unit of years) by aging clock microarray analysis of one‐time IOP‐treated 3‐month and 18‐month retinae and multiple IOP‐treated 3‐month retinae. *n* = 3 biological replicates. Statistical significance determined using unpaired t‐test. Mean and SD are shown. *p < 0.05; **p < 0.01; ***p < 0.001. (b) Volcano plots representing the significant changes in CpGs methylation genome‐wide, on promoters and enhancers. Each dot represents one CpG site with −log10(adjusted *p*‐value) versus log2foldchage. The significance of change in CpG methylation was defined with cutoffs at 0.05 in adjusted *p*‐value and 0.5 in log2foldchange. (c) Volcano plot showing the distribution of significantly upregulated CpGs and the number of downregulated genes associated with them. Pie chart – % of downregulated genes reversely correlated with CpGs changes relative to total number of genes with significantly upregulated methylation levels of CpGs in their regulatory regions. (d) Volcano plot showing the distribution of significantly downregulated CpGs and the number of upregulated genes associated with them. Pie chart – % of upregulated genes reversely correlated with CpGs changes relative to total number of genes with significantly downregulated methylation levels of CpGs in their regulatory regions. (e) Venn diagrams showing the number of genes that have both regulatory elements (promoters and enhancers) changed in correlation with the gene expression. The expression pattern of the final group of genes does not reversely correlate with the changes on CpGs located in deregulated regulatory regions. (f) GO term analysis of genes whose expression reversely correlates with significantly lowered methylation of CpGs in regulatory regions.

Next, we asked whether repeated IOP elevation in young retinas can induce acceleration of aging according to DNA methylation‐based estimators of age (“epigenetic clocks”). We isolated DNA from the control and 4xIOP retinas. We used the Mammalian Methylation 320 k Array designed for mouse methylation studies (Arneson et al., 2022; Lu et al., 2021). We used previously published pan‐tissue mouse clock as well as tissue specific clocks for liver, blood, brain, muscle, heart, cortex, striatum, cerebellum, tail, kidney, skin, and fibroblasts (Mozhui et al., 2022). Since there is no retina‐specific DNA methylation clock, our primary analysis focused on the PanTissue mouse clock and the universal pan mammalian clock that applies to all mammalian species (Lu et al., 2021). The resulting epigenetic age estimates are strongly associated with age (3‐month‐old versus 18‐month‐old, Figure 6a). The same clocks detected that 3‐month‐old tissue after 4xIOP stimuli exhibits increased DNA methylation age in the retina. Furthermore, DNA methylation age was greatly increased in repeatedly stressed 3‐month‐old tissue coming close to that of 18‐month‐old retina. Similar correlation was observed using the mouse brain methylation clock. As expected, epigenetic clocks that were not designed for brain tissue (such as the blood clock) were not able to detect differences in age between young and old tissues as well as young and 4xIOP‐treated tissues (Figure 6a). Taken together, DNA methylation data confirm the aging effects detected using transcriptional data; however, it underlines the need of using adequate epigenetic clocks.

Next, we sought to explore whether the pattern of DNA methylation can indicate specific pathways involved in retina aging. To do that, we correlated DNA methylation marks with RNAseq data from young and old retinas. The differential analysis showed that a total of 61 CpG sites had increased while 1203 had decreased levels of DNA methylation in aged versus young retinas (*N* = 4) with significance cutoffs at 0.5 in log2 foldchange (about 1.4‐fold) and adjusted *p*‐value <0.05. (Figure 6b). Among the 61 up‐regulated CpG sites in DNA methylation, 6 were located at promoter regions (6 unique genes) while 29 were located at enhancer (intron and intergenic) regions (28 unique genes). Among the 1203 down‐regulated CpG sites in DNA methylation, 102 were located at promoter regions (91 unique genes), while 858 were located at enhancer (intron and intergenic) regions (615 unique genes). (Figure 6b).

Combining aging‐related DNA methylation changes with aging‐related differential expression from RNA‐seq analysis, we detected: 3 regulatory regions with increased CpG methylation correlating with downregulated genes (Figure 6e), and 146 regulatory regions with lowered CpG methylation located in proximity to 102 upregulated genes (Figure 6e). We used the latter group to perform EnrichR analysis and noticed highly significant enrichment of “negative regulation of transcription” and Tgfβ pathways (Figure 6f). RNAseq data confirmed their expression of selected genes in aged in comparison with young retina (Figure S6).

## 3 DISCUSSION

In this work, we focused on understanding differences in stress response between young and old animals in an experimental model of ocular hypertension. First, we describe a significant decline in visual function with age. These changes correlate with transcriptional changes in the retina, showing increase of inflammatory and cell death pathways, as well as degradation of ECM integrity during aging. Our epigenetic analysis has shown an increase in chromatin accessibility with age, mostly on enhancers. Next, we show that upon mild IOP elevation, aged retina further activates inflammatory, senescence, and other age‐related pathways and that these changes occur also in RGCs. Interestingly, the response to the same, mild stress, in young tissue is much weaker and does not trigger RGC death. The high response in aged animals correlates with substantial increase of H3K27Ac on regulatory regions of upregulated genes. Finally, we show that we can induce very strong stress response in young tissue upon mild hypertension once the retina is “primed” with previous instances of mild IOP elevation. We conclude that several occurrences of stress in young retina accelerate features of aging such as loos of vision and, on molecular level, transcription profile and by DNA methylation clock.

Glaucoma is characterized by slow, progressive loss of RGCs, sectorial progression in optic nerve degeneration, and eventual vision loss (Weinreb et al., 2014). Here, we characterize new animal model relevant to the human disease that can allow study of the time course of molecular events in the retina upon mild ocular hypertension. We show that 18‐month‐old animals loose RGCs and vision as measured by PERG upon 30 mmHg IOP elevation for 1 h, while 3‐month‐old animals do not show these phenotypes. In addition, cross‐section of optic nerve of the treated and non‐treated eyes shows sectorial loss of axons but only in old animals. Finally, our molecular analysis detects senescence as one of the pathways involved in the response to stress mechanism of the retina upon IOP elevation, what we previously documented in human glaucomatous tissue (Skowronska‐Krawczyk et al., 2015).

A main hurdle in developing new therapies and understanding the molecular mechanism of glaucoma is the lack of an animal model that recapitulates all aspects of the disease. While some laboratories focus on developing animal models of chronic IOP elevation, others use mice with natural mutations that slowly develop age‐related hypertension and RGC loss (Di Pierdomenico et al., 2022). We have been particularly interested in studying an animal model that allow us to capture the time course of molecular changes upon glaucomatous stress, such as mild ocular hypertension. Since age is the strongest risk factor for developing glaucoma, we developed and characterized the aged animal model for our future molecular studies.

The molecular analysis of transcriptional changes between IOP‐treated and non‐treated retinas has detected a high number of pathways involved in innate immunity and sterile inflammation. The significant changes can be observed in aged animals; however, most of the same genes are also slightly upregulated in 3‐month‐old animals without reaching significance. Interestingly, a similar coordinated inflammatory program was observed when we compared transcriptional differences between young and old retina transcriptomes suggesting that IOP stress was able to elicit age‐related inflammatory response in the tissue. In fact, the list of pathways upregulated in aged and aged‐IOP‐treated retinas is similar and analogous to the set of pathways called “inflammaging” in previous studies (Franceschi et al., 2018). Since RGCs degenerate early in glaucoma, we sought to find whether observed transcriptional changes happen in RGCs, as well. We observed that upon 30 mmHg IOP elevation, RGCs express hallmarks of senescence: the cell cycle inhibitors *p16Ink4a* and *p19Arf* (Figure 3b, Figure S3), as we have previously shown in human glaucomatous retinas. These observations further confirm the ability of neurons, postmitotic cells, to assume senescent phenotype (Oubaha et al., 2016; Skowronska‐Krawczyk et al., 2015; Von Zglinicki et al., 2021). Our data also show that several factors from other pathways, such as oxidative stress, immune response, extracellular matrix homeostasis, and inflammation are upregulated in GCL (Figure 3a–c, and Figure S3), show that these neurons dynamically respond to the stress signal. Not surprisingly, given the paracrine nature of senescence, other cell types also express several of the tested genes. Interestingly *Tgfβ*, *Fas*, and FGF2 are almost exclusively upregulated in RGCs, once again suggesting that these cells are directly responding to stress and suggesting that the observed epigenetic changes occur in RGCs. At this point, we cannot know which cell types respond first to the stress and where the epigenetic changes start.

Moreover, we show, even in aged retina, that RGCs can be protected from ocular hypertension‐related death with senolytic drugs that remove senescent cells (Figure 3d). These data suggest that senescence and a set of inflammatory genes are pre‐programmed to swiftly respond to stress in aged retina. This is why we asked whether changes in DNA accessibility can explain the different levels of response to the same signal between young and old tissue.

The chromatin accessibility assay data are puzzling. During the 15 months of retina aging, only ~250 sites in the chromatin opened, most of them being enhancers (Figure 1h). Even fewer sites lost their accessibility (~70). Given the fact that one gene can have several regulatory elements and there are ~4000 deregulated transcripts in aging (Figure 1d), we assume that most of the regulation of the expression of the age‐related changed transcripts is not regulated at the level of DNA accessibility. In fact, the chromatin accessibility status seems to be in principle set already at 3‐moth‐old animal. In the mild IOP data sets (RNAseq vs. ATAC‐seq), the numbers of transcripts are similar to numbers of detected open chromatin sites; however, we could not detect correlation of upregulation of expression and increase in enhancer accessibility in these samples (Figure 4e) suggesting other pathways being involved in regulation of activation of expression of these enhancers. H3K27Ac histone mark is traditionally associated with active regulatory elements. We tested whether there are differences in dynamics in levels of this chromatin modification upon stress in young and old retinas. Indeed, in old tissues, H3K27Ac levels increased, suggestive of activation of enhancers and promoters. In contrast, in young tissues, the levels of H3K27Ac dropped, suggesting that specific lack of the activation of expression of these genes in 3‐month‐old animals is associated with active repression of their regulatory elements. Further studies are needed to address this fascinating point.

After looking at the conserved TF binding sites on open regions, we have detected that STAT factors were particularly highly represented (Figure S4B). Interestingly, on the list of the TF binding sites on open regions in stressed young and aged retinas, we noticed binding sites of homeodomain TFs, such as Crx and Otx2, detected previously as enriched in sites opened in natural aged retina (Figure 1I). This observation further shows similarities between the stress response and aging.

Following these findings, we asked whether repetitive mild stress in young animals can induce accumulation of stress response and tissue damage. We allowed two weeks of recovery between each 30 mmHg IOP elevation (Figure 5a). Transcriptomic data were clear – retinas that underwent recurrent mild stress exhibited significant upregulation of almost 900 genes (Figure 5b). GSEA analysis indicated high upregulation of inflammatory pathways and deregulation of apical junction (Figure S5A). Metascape analysis clustered all pathways in inflammation‐related bundles, highlighting the level of sterile inflammation induced upon repeated stress (Figure S5B). The response to recurrent stress was significantly higher than in the 3‐month‐old retina treated one time. In fact, the transcriptional response of the 4xIOP‐treated retina was very similar to that of the 18‐month‐old retina upon a single 30 mmHg IOP elevation (Figure 5d, Figure S5B). Therefore, the transcriptional phenotype suggested that the tissue aged with several rounds of stress.

DNA methylation‐based clocks were developed around 10 years ago, to objectively and independently, assess the chronological age of the human, animal, tissue, and any nucleated cells (Garagnani et al., 2012; Hannum et al., 2013; Weidner et al., 2014). As DNA methylation levels are strongly correlated with age, the profile of genome‐wide CpG methylation status can be used to estimate the age of a specimen. Interestingly, it has been shown that although similar, each tissue has its own pace of DNA methylation change and, therefore, one can develop a specific clock for any given tissue (Horvath, 2013; Zhang et al., 2013; Zhou et al., 2017). These “DNA methylation clocks” can be then used to follow the impact of diseases or environmental cues on aging (Horvath & Raj, 2018). They have been shown to be especially useful in population genetic studies by uncovering new associations between aging and different health conditions (Levine et al., 2015; Weidner et al., 2014). As there is no mouse retina‐specific clock, we decided to work with pan tissue mouse clocks, brain clocks, and universal pan mammalian clocks developed by the Horvath laboratory (Lu et al., 2021; Mozhui et al., 2022). Importantly, due to the lack of a retina‐specific clock, the numbers of years (age of tissue) obtained in the assay should be treated as relative levels. Strikingly, both the PanTissue and Universal clocks showed that repeated IOP stress strongly accelerates DNA methylation age (Figure 6a). Moreover, 4xIOP tissue was now closer, epigenetically, to the 18‐month‐old DNA methylation age. Thus, both transcriptomic and DNA methylation data collectively imply that multiple instances of mild IOP can accelerate aging of the retina. Our further analysis (Figure 6b–d) attempting to correlate gene expression with DNA methylation has shown that genes involved in negative regulation of transcription and *Tgfβ* pathways are upregulated in aged tissues while DNA methylation on their regulatory elements is increased (Figure 6f,g). Since we used pan‐tissue epigenetic clocks in this analysis, which are expected to be agnostic to the tissue, this analysis suggests that the tissue‐independent genes involved in aging are related to downregulation of transcription and the TGFβ pathways.

In other studies, researchers attempted to “rejuvenate” retina. Upon the overexpression of Yamanaka factors, they could reverse the DNA methylation aging signature in the context of optic nerve injury, glaucoma model in mice and natural aging (Lu et al., 2020). This shows not only the reversibility of the, previously believed, “stable” DNA methylation but also possibility of potential treatments. This has been also shown in other studies using the reprogramming approaches (Browder et al., 2022; Lu et al., 2020; Ocampo et al., 2016; Sarkar et al., 2020). Our work, for the first time, indicated the direct impact of stress on chromatin modifications and their genome‐wide localization, therefore suggesting another modifiable level of transcription regulation. Remining question of whether the DNA methylation and histone modification patterns are stable after the impact of repetitive mild stress will require further investigations.

In humans, IOP has a circadian rhythm (Liu et al., 1999). In healthy individuals, it oscillates typically in the 12–21 mmHg range and tends to be highest in approximately two‐thirds of individuals during the nocturnal period (Mosaed et al., 2005). Due to IOP fluctuations, a single IOP measurement during so‐called office hours is often insufficient to characterize the real pathology and risk of disease progression in glaucoma patients. Long‐term IOP fluctuation has been reported to be a strong predictor for glaucoma progression, especially in individuals with low mean IOP levels (Kim & Caprioli, 2018). It has been also suggested that dysregulation of the diurnal IOP cycle, with higher daily amplitudes and longer periods of ocular hypertension, might be the cause of some cases of glaucomatous optic neuropathy (Aptel et al., 2016). Since IOP at a level sufficient to cause ischemia is significantly more severe than even the highest circadian fluctuations in glaucoma patients, it is critical to study glaucoma‐related phenomena in the models more closely recapitulating human disease. In this study, we have shown that even moderate hydrostatic IOP elevation to 30 mmHg for 1 h, a level relevant to human disease, results in RGC loss and corresponding visual defects when performed on aged animal.

It is well established that scleral elasticity decreases with age; however, it is not known whether changes in scleral elasticity with age contribute to optic nerve damage. In mice, it has been reported that IOP does not increase in C57B/6J animals with age (Savinova et al., 2001) and from our data in we conclude that the function and RGC numbers are not substantially affected by age (Figure 1). In addition, our repetitive IOP experiments in young animals showing that stress‐induced accelerates aging in the retina suggest that age‐related changes in sclera are not key to cause the phenotype. It is however not excluded that impact of higher IOP in aged animals is partially amplified by the lower scleral elasticity, further emphasizing the importance of using aged animals when studying age‐related eye disease such as glaucoma.

### 3.1 Conclusions

In sum, we have shown that susceptibility to stress changes with age and is pre‐conditioned epigenetically. Amongst the pathways activated upon IOP elevation in 18‐month‐retina are inflammation and senescence. Moreover, the use of a senolytic drug can protect RGCs from death. The robust response in aged retina was primed by epigenetic changes which we were able to reproduce by performing repetitive stress on the young tissue. Finally, we have shown that multiple instances of stress in 3‐month‐old animal cause changes in transcriptional and DNA methylation patterns indicating accelerated features of aging. This process was accompanied by gain of activatory histone mark on regulatory elements of upregulated genes implying that molecular mechanism of aging is regulated at the level of chromatin modification. This in turn suggests that rate of aging may be modifiable using drugs affecting enzymes involved in chromatin modifications. Finally, these results emphasize the importance of early diagnosis and prevention as well as age‐specific management of age‐related eye diseases, including glaucoma.

Our studies are also an example of potential use of retina, an integral part of the central nervous system (CNS), as a model system highly suitable to study molecular and functional changes in CNS aging and in neurodegenerative disorders (Wareham et al., 2022). The retina is highly organized, well‐described, and highly accessible. The availability of technologies to measure and directly visualize the stepwise process of degeneration including well‐developed methods to measure visual functions further highlights high value of studies in the eye for the understanding of neurodegeneration. Furthermore, retina provides an easy and highly accessible way to visualize age and degeneration‐related events in CNS. For example, analysis of images of the patients' back of the eyes have been shown to correlate or predict different health‐related phenomena such as Alzheimer's disease and chronic kidney disease (Chiquita et al., 2019; Paterson et al., 2020). Surprisingly, AI analysis of retinal images has been also shown to reliably predict cardiovascular risk factors such as age, gender, smoking status, and systolic blood pressure (Poplin et al., 2018). We therefore argue that studying mouse models of age‐related eye diseases can provide invaluable information in understanding and treatment development for other neurodegenerative disorders.

## 4 MATERIALS AND METHODS

### 4.1 Hydrostatic intraocular pressure (IOP) elevation

Animals were anesthetized with an intraperitoneal injection of ketamine/xylazine cocktail, (100 mg/kg and 10 mg/kg, respectively), their eyes numbed with a drop of proparacaine (0.5%, Bausch‐Lomb) and dilated with a drop of tropicamide (1%, Alcon Laboratories) followed by a drop of phenylephrine (2.5%, Akorn Pharmaceuticals). To achieve IOP elevation, a 33‐gauge needle was advanced trans corneally into the anterior chamber under visual control. Elevation of IOP was achieved by instilling the aqueous chamber of the eye with Balanced Salt Solution (Alcon Laboratories) through an IV infusion set. The desired level of IOP increase was achieved by pumping infusion bag under control of sphygmomanometer. Stable elevated IOP was maintained for 60 minutes, controlled by IOP measurements using a veterinary rebound tonometer (Tonovet). Both eyes were lubricated with an ophthalmic lubricant gel (Alcon Laboratories) during the protocol. Following the procedure, one drop of topical antibiotic was applied to the treated eye (Ofloxacin 0.3%, Apexa). The anesthesia was reversed with Atipamezole (0.1 mg/kg) and animals recovered on an isothermal pad until awake. The contralateral eye without IOP elevation served as a healthy non‐IOP control (CTRL).

Animals were treated at 3 pressure levels (30, 50, and 90 mmHg), and studied at two timepoints (7 and 21 days).

To control for procedural effects, two additional pressure level cohorts—7 mmHg, and 15 mmHg—were investigated at the day 7 time point. The 7 mmHg eyes were cannulated without elevating pressure through the IV infusion set. Each experimental group consisted of *n* = 6 animals (3 male, 3 female). Additionally, we studied a cohort of 6‐month‐old mice (3 male, 3 female) 7 days after following unilateral hypertension to 30 mmHg for 1 h.

### 4.2 Neurophysiology

Animals were initially anesthetized with 2% isoflurane in a mixture of N_2_O/O_2_ (70%/30%) then and placed into a stereotaxic apparatus. A small, custom‐made plastic chamber was secured to the exposed skull using dental acrylic. After one day of recovery, re‐anesthetized animals were placed in a custom‐made hammock, maintained under isoflurane anesthesia (1%–2% in N_2_O/O_2_), a craniotomy was performed, and multiple single tungsten electrodes were inserted into V1 layers II‐VI. Following electrode placement, the chamber was filled with sterile agar and sealed with sterile bone wax. Animals were then sedated with chlorprothixene hydrochloride (1 mg/kg; IM) and kept under light isoflurane anesthesia (0.2%–0.4% in 30% O_2_) throughout the recording procedure. EEG and EKG were monitored throughout, and body temperature was maintained with a heating pad (Harvard Apparatus; Figure 1).

Data were acquired using a multi‐channel Scout recording system (Ripple, UT, USA). Local field potentials (LFP) from multiple locations at matching cortical depths were band‐pass filtered from 0.1 to 250 Hz and stored along with spiking data at a 1 kHz sampling rate. LFP signal was aligned to stimulus time stamps and averaged across trials for each recording depth in order to calculate visually evoked potentials (VEP; Foik et al., 2015). Single neuron spike signals were band‐pass filtered from 500 to 7 kHz and stored at a 30 kHz sampling frequency. Spikes were sorted online in Trellis (Ripple) while performing visual stimulation. Visual stimuli were generated in Matlab (Mathworks) using Psychophysics Toolbox and displayed on a gamma‐corrected LCD monitor (55 inches, 60 Hz; 1920 × 1080 pixels; 52 cd/m^2^ mean luminance). Stimulus onset times were corrected for monitor delay using an in‐house designed photodiode system (Foik et al., 2018). Visual responses were assessed according to previously published methods (Foik et al., 2018; Foik et al., 2020). For recordings of visually evoked responses, animals were tested with 100 repetitions of a 500 ms bright flash of light (105 cd/m^2^).

### 4.3 Local field potential (LFP) analysis

Amplitude of response was calculated as a difference between the peak of the positive and negative components of the VEP. Response latency was defined as the time from stimulus onset to maximum response. Maximum of the response was defined at the larger of the negative or positive peak.

### 4.4 Visual evoked potential (VEP)

VEP measurements were recorded at 7 and 21 days post‐IOP elevation according to a previously published protocol. Mice were dark adapted for at least 12 hours before the procedure. Animals were anesthetized, their eyes dilated as in the IOP elevation protocol. The top of the mouse's head was cleaned with an antiseptic solution. A scalpel was used to incise the scalp skin, and a metal electrode was inserted into the primary visual cortex through the skull, 0.8 mm deep from cranial surface, 2.3 mm lateral to the lambda. Platinum subdermal needle (Grass Telefactor) was inserted through the animal's mouth as reference, and through the tail as ground. The measurements commenced when baseline waveform became stable, 10–15 s after attaching the electrodes. Flashes of light at 2 log cd.s/m^2^ were delivered through a full‐field Ganzfeld bowl at 2 Hz. Signal was amplified, digitally processed by the software (Veris Instruments), then exported and peak‐to‐peak responses were analyzed in Excel (Microsoft). To isolate VEP of the measured eye from the crossed signal originating in the contralateral eye, black aluminum foil eyepatch was used to cover the eye not undergoing measurement. For each eye, peak‐to‐peak response amplitude of the major component N1 in IOP eyes was compared to that of their contralateral NIOP controls. Following the readings, the animals were euthanized, their eyes collected and processed for immunohistochemical analysis (Figure 2 and Figure S2).

### 4.5 Optomotor responses

Optomotor responses (OMRs) were recorded using commercially available qOMR setup. (PhenoSys GmbH, Berlin, Germany). The software automatically tracks animal head movement in relation to the moving grating stimulus and calculates correct/incorrect tracking behavior presented as quantitative optomotor index (qOMR). The mouse was placed on elevated platform in OMR arena. Grating stimuli (rotating at 12°/s) were presented for ~12 min per trial. The spatial frequency of the grating was set at 0.2 cycles per degree of visual angle. The stimulus was presented at differing contrasts between the light and dark: 3, 6, 10, 12.5, 15, 17.5, 20, 25, 37.5, 50, 75, 100 contrast. Stimulation at each contrast level lasted 60 s. Each mouse was tested in 4 photopic trials and 4 scotopic trials. Each mouse was firstly tested in photopic conditions and then in scotopic. For the scotopic part of the experiment (resembles nighttime light levels), the animals were dark‐adapted for 12 h before the experiments. The OMR arena was dimmed using density 4 filters in front of the stimulus displays. The luminance for scotopic part was ~0.03 lux. Results for 100% contrast were excluded due to adjustment (mice adjustment to the system). qOMR results that yielded correct/incorrect ratio lower than 0.8 were excluded. Results from the trials were averaged for analysis of each individual mouse. Each data point represents average of all mice tested. GraphPad prism was used for statistical analysis and to generate curves.

### 4.6 Pattern electroretinography

All mice were dark‐adapted for 12 h before pattern electroretinography (PERG) was recorded. PERG recordings were obtained with the commercially available Celeris ERG platform equipped with pattern ERG stimulators (Diagnosys LLC). Prior to experiment, all mice were anesthetized with Ketamine/Xylazine (100 mg/kg and 10 mg/kg, respectively) injected intraperitoneally.

The eyes were anesthetized with proparacaine (0.5%, Bausch‐Lomb); next, the eyes were dilated using phenylephrine (2.5%, Akorn Pharmaceuticals) and tropicamide (1%, Alcon Laboratories) commercially available eyedrops. The eyes were lubricated with corneal gel. After anesthesia and eye dilation pattern, erg stimulators were placed on corneal surface. For all mice, 400 sweeps (reads) per eye were recorded. PERG amplitude was measured between first positive peak (P1) and second negative peak (N2).

### 4.7 Senolytic drug treatment

The experimental group of animals was treated by intraperitoneal (IP) administration of dasatinib (Sigma, 5 mg/kg) after IOP elevation (see below), and a control group of mice was sham‐treated vehicle (PBS/DMSO). Each mouse underwent unilateral hydrostatic pressure‐induced IOP elevation to 30 mm Hg, with the contralateral eye left as an untreated control. The mice were IP injected intraperitoneally with dasatinib at day 0 (IOP elevation day) and continued for four consecutive days (Figure 3d). At day 5, animals were euthanized, and retinas were isolated and immunostained with anti‐Brn3a antibody to evaluate the number of RGCs. All drugs were prepared according to the UC Irvine Institutional Animal Care and Use Committee (IACUC) standards. To ensure a sterile environment, compounds were prepared under the tissue culture hood using sterile PBS. The final solution was filtered through a 0.22‐μm PES membrane just before injection. Tips, tubes, and syringes were sterile.

### 4.8 Immunohistochemistry for RGC count (anti‐Brn3a and anti‐RBPMS)

Eyes were fixed in 4% paraformaldehyde (PFA) in PBS for 2 h and then transferred to PBS. The retinas were extracted through dissection and were flat‐mounted on microscope slides and blocked for 1 h in blocking solution (10% bovine serum albumin [BSA] diluted in 0.5% Triton‐X/PBS). Standard sandwich assays were performed to immunostained using anti‐Brn3a antibodies (Millipore, MAB1585) and anti‐RBPMS antibodies following secondary Alexa Fluor 555 anti‐mouse (Invitrogen, A32727) and secondary Alexa Fluor 647 anti‐rabbit (Invitrogen, A21245). The samples were then mounted (ProLong Gold antifade reagent, Invitrogen P36934) and imaged using a fluorescent microscope at 20x magnification (Biorevo BZ‐X700, Keyence). Peripheral retina regions were chosen to optimize the quality of the micrographs. The micrographs, which were 0.39 mm^2^ per region, were split into 4 equal parts to exclude disqualified, damaged regions. Labeled cells were manually quantified with the “cell counter” function of the software Fiji.

### 4.9 Optic nerve head—tissue preparation and immunohistochemistry

Mice were anesthetized by an IP injection of a cocktail of ketamine/xylazine as described above prior cervical dislocation. For immunohistochemistry, the optic nerve head (ONH) tissues were dissected from the eyeballs and fixed with 4% paraformaldehyde (Sigma) in phosphate‐buffered saline (PBS, pH 7.4, Sigma) for 2 h at 4 °C. ONHs were washed several times with PBS then dehydrated through graded levels of ethanol and embedded in polyester wax.

Immunohistochemical staining of 7 μm polyester wax sections of full thickness retina was performed. Sections from polyester wax blocks from each group (*n* = 3 ONHs/group) were used for immunohistochemical analysis. To prevent non‐specific background, tissues were incubated in 1% bovine serum albumin (BSA, Sigma)/PBS for 1 h at room temperature before incubation with mouse monoclonal NF68 antibody for 16 h at 4°C. After several wash steps, the tissues were incubated with the Alexa Fluor‐488 conjugated donkey ant‐mouse IgG antibody for 4 h at 4°C and subsequently washed with PBS. The sections were counterstained with the nucleic acid stain Hoechst 33342 (1 μg/ml; Invitrogen) in PBS. Images were acquired with Keyence All‐in‐One Fluorescence microscopy (BZ‐X810, Keyence Corp. of America). NF68 protein fluorescent integrated intensity in pixel per area was measured using the ImageJ software. All imaging parameters remained the same and were corrected with the background subtraction.

### 4.10 RNA‐sequencing (next generation sequencing)

Fresh retina tissue of each mouse eye was homogenized thoroughly in TRIzol Reagent (Cat no.15596026, Invitrogen) followed by RNA isolation. For pellet of immunoselected RGCs—cells were resuspended in TRIzol reagent. The polyadenylated (poly‐A) transcripts from the isolated total RNA sample were enriched and performed strand‐specific mRNA‐Seq libraries preparation by TruSeq kit followed by next‐generation sequencing on NovaSeq 6000 System (Flow Cell Type S4). mRNA‐Seq raw reads data was mapped to mm10 assembly mouse genome by homer (v4.11) aligner tool STAR. The uniquely mapped tags in exons on minus strand (one isoform per locus) were quantified by analyzeRepeats.pl. Raw reads count was normalized to experiment totals and performed differential expression based on the negative binomial distribution by DESeq2 v1.24.0 program (Love et al., 2014) with FDR q‐value significance cut‐off at 0.05 and log2(fold change) significance cut‐off at 1 for up‐regulation or −1 for down‐regulation.

### 4.11 Gene set enrichment analysis (GSEA)

The GSEA v4.2.3 was performed for pairwise differential expression comparisons between two phenotype groups of RNA‐Seq samples in the pre‐ranked module (Subramanian et al., 2005). The target chip annotation platform was Mouse_RefSeq_Accession_Extended_Human_Orthologs_MSigDB.v7.5.1. chip and gene set collection applied was the condensed hallmark pathways (v7.5.1) from the Molecular Signatures Database (MSigDB; Liberzon et al., 2015). The differential expression metrics were ranked by Rank Metric Score (Log2Fold Change * Log10(FDR *q*‐value)). Enrichment analysis was performed using weighted (*p* = 1) scoring calculation scheme with 1000 permutations. Gene sets with size larger than 500 and smaller than 5 were excluded from the analysis.

### 4.12 Metascape gene enrichment and functional analysis

List of RefSeq identifiers of differential expressed (DE) genes (FDR *q*‐value ≤0.05; log2foldchange ≥1 as significant up‐regulated DE genes or ≤−1 for downregulated) from transcriptome analysis of each comparison pair (Zhou et al., 2019) were abundantly annotated by retrieving from multiple databases. The given list of annotated gene identifiers was then statistically enriched into terms by selected ontology sources which contain Gene Ontology Biological Processes, KEGG Pathways, Reactome Gene Sets, and WikiPathways. Terms with a *p*‐value <0.01, a minimum count of 3, and an enrichment factor >1.5 (the enrichment factor is the ratio between the observed counts and the counts expected by chance) are collected and grouped into clusters based on their membership similarities. To further capture the relationships among the terms, the subsets of the enriched terms were rendered as interaction networks where terms with a similarity >0.3 are connected by edges and formed intra‐ and inter‐clusters The Metascape enrichment networks were finally visualized in nodes‐and‐edges by Cytoscape (v3.9.0). Node size is proportional to the number of input genes in the term. Each node represents an enriched term and was either colored by cluster ID or colored in discrete scale by FDR q‐value.

### 4.13 ATAC‐sequencing

Fresh retina tissue of each mouse eye was re‐suspended immediately in 1 ml ice‐cold nuclei permeabilization buffer (5% BSA, 0.2% [m/v] NP40, 1 mM DTT in PBS solution) with 1X complete EDTA‐free protease inhibitor and homogenized using a syringe and needle followed by slow rotation for 10 min at 4°C. The nuclei suspension was then filtered through a 40uM cell strainer and centrifuged for 5 min at 500× *g* at 4°C. The nuclei pellet was resuspended in an ice‐cold 50 μl tagmentation buffer. Nuclei concentration was adjusted to 2000–5000 nuclei/μl and 10 μl of the suspension was used for tagmentation. 0.5 μl Tagment DNA Enzyme 1 (FC‐121‐1030, Illumina) was added to the 10uL suspension. The reaction mix was thoroughly pipetted and incubated 30 minutes with 500 rpm at 37°C. After the tagmentation reaction was completed, the DNA was isolated using Qiagen PCR Purification Kit (Cat.#0.28304, Qiagen) and elute in 20 μl Elution Buffer. The eluted DNA fragments were then amplified by PCR with Nextera compatible indexed sequencing i5 and i7 adapters using NEBNext 2x PCR Master Mix PCR kit (M0541, NEB). The amplified DNA library was fragment size selected from 200 to 800 bp using Ampure XP beads (A63880, Beckman Coulter). The quality of the ATAC‐Seq libraries was assessed by Agilent 2100 bioanalyzer (Agilent Technologies, Inc.). ATAC‐Seq libraries were pooled and run on a NovaSeq 6000 System (Flow Cell Type S4) Illumina sequencer with a paired‐end read of 100 bp to harvest about 25 million paired‐end reads per sample.

### 4.14 ChIP‐seq

Fresh retina tissue of each mouse eye was homogenized with 22Gx1′ BD Precision‐glide syringe‐needle and crosslinked by 1%(w/v) methanol‐free formaldehyde (Cat.no.28906, Thermo Scientific) in crosslinking buffer (10 mM HEPES‐NaOH pH 7.5, 100 mM NaCl, 1 mM EDTA, 1 mM EGTA) for 10 min slow rotation at room temperature. Crosslinks were quenched by glycine with 125 mM final concentration. Tissue samples were lysed into chromatin with buffer 1 (0.25% Triton X‐100, 0.5% NP40, 10% glycerol in crosslinking buffer). 10 mM sodium butyrate was added for deacetylation inhibition. Lysed tissue was pelleted and washed once with buffer 2 (200 mM NaCl in 10 mM. Tris–HCl pH 8.0). Chromatin samples were sonicated (Pico Bioruptor, Diagenode) in polystyrene tubes on low power with 8 cycles of 30 s “on,” 45 s “off” in buffer 3 (10 mM Tris–HCl pH 8.0, 100 mM NaCl, 1 mM EDTA, 0.5 mM EGTA, 0.1% sodium deoxycholate and 0.5% N‐lauroylsarcosine) and further cleared from debris by 16,000 g centrifuge. Anti‐Histone H3 acetyl K27 antibody (Cat no. ab4729, Abcam) was coupled with protein A Dynabeads (Life Technologies) in blocking buffer (0.1% BSA, 1% Triton X‐100) before 16 hours incubation with beads precleared chromatin at 4°C. Dynabeads with antibody‐enriched chromatin were washed twice with low salt wash buffer (150 mM NaCl, 2 mM EDTA, 0.1%SDS, 1%Triton X‐100 in 20 mM Tris–HCl pH 8.0) and twice with lithium chloride wash buffer (1 mM EDTA, 0.5% Sodium Deoxycholate, 0.25 M LiCl, 1% NP40 in 10 mM Tris–HCl pH 8.0). Beads were finally incubated at 65°C in TE with 1% SDS at 65°C for 16 h with shaking for decrosslinking of the enriched chromatin. Chromatin DNA was isolated using Qiagen PCR Purification Kit (Cat.No.28304, Qiagen) and elute in 20 μl Elution Buffer. The eluted DNA fragments were then amplified by PCR with Nextera compatible indexed sequencing i5 and i7 adapters using NEBNext 2x PCR Master Mix PCR kit (M0541, NEB). The amplified DNA library was fragment size selected from 200 bp to 800 bp using Ampure XP beads (A63880, Beckman Coulter). The quality of the ChIP‐Seq libraries was assessed by Agilent 2100 bioanalyzer (Agilent Technologies, Inc.). ChIP‐Seq libraries were pooled and run on Illumina NovaSeq 6000 System (Flow Cell Type S4) sequencer with a paired‐end read of 100 bp to harvest about 25 million paired‐end reads per sample.

### 4.15 DNA methylation clocks analysis

The Horvath Mammalian Methylation 320 k Chips designed for mouse methylation studies (Arneson et al., 2022) were used. This enables the quantitative interrogation of more than 320,000 CpGs per sample, including >285,000 specific to mice and 37,000 conserved mammalian loci. 250 ng DNA of each sample (*n* = 4 for each group) underwent DNA extraction, bisulfite conversion, DNA methylation arrays, and statistical analysis as previously described (Lu et al., 2021). Testing was performed for the pan‐tissue mouse clock as well as for liver, blood, brain, muscle, heart, cortex, striatum, cerebellum, tail, kidney, skin, and fibroblasts.

### 4.16 RNA‐Scope and HiPlex

In situ hybridization was performed using the RNAscope® Multiplex Fluorescent Assay v2 (ACD Diagnostics) and (HiPlex) assay (HiPlex 12 Reagents Kit, ACD Diagnostics). Probes used were designed by the manufacturer (see Table 1). Briefly, fresh frozen histologic sections of mouse eyes were pretreated per manual using hydrogen peroxide and target retrieval reagents including protease IV. Probes were then hybridized according to the protocol and then detected with TSA Plus® Fluorophores fluorescein, cyanine 3, and cyanine 5. Sections were mounted with Prolong Gold Antifade (Thermo Fisher) and imaged (Keyence BZ‐X700).

**TABLE 1:** Catalog numbers of antibodies and RNA probes used in experiments.

Antibody/probe name	Catalog number	Vendor
Antibodies		
Anti‐CD87 (UPAR) antibody, PE	MA5‐28588	Invitrogen
Anti‐FGF‐2 antibody (G‐2)	SC‐365106	Santa Cruz Biotech
Anti‐Histone H3 (acetyl K27)	ab4729	Abcam
RNAscope Probes		
p16Ink4a (Cdkn2a‐tv2‐p16)	447491‐C3	Advanced Cell Diagnostics Inc
p19Arf (Cdkn2a‐p19)	411011‐C2	
mm‐Rbpms	527231	
HiPlex probes		
Tgfb1	1071891‐T3	Advanced Cell Diagnostics Inc
Fas	439561‐T6	
Hmox1	498811‐T7	
Cd44	476201‐T8	
Ccl2	311791‐T10	
Fgf2	316851‐T12	

## AUTHOR CONTRIBUTIONS

DS‐K with help from QX—study conceptualization and design; QX—next‐gen experiments performance and genome‐wide data analysis; CR, VANH, LR, CPLT—animal experiments; IL—immunostaining, RGC quantification; WC—immunostaining, RNA‐scope experiments; MJ—molecular experiments; JD—optic nerve experiment quantification, lab assistance; RNW—expert data analysis and manuscript editing; DL and AF—VEP experiments data acquisition, analysis and figures preparation; BB and SH—DNA methylation data acquisition and analysis; W‐KJ—optic nerve‐head experiments, expert manuscript editing; DS‐K, QX and CR—figure preparation and manuscript writing; all authors read, edited and gave final approval of the manuscript to be published.

## 5 ACKNOWLEDGEMENTS

The authors wish to thank Dr. Henri Leinonen for help in establishing the OMR methods, Vincent Duc‐Vinh Mai for help with mouse experiments and all members of DSK laboratory for helpful discussions.

## FUNDING INFORMATION

Work in Dorota Skowronska‐Krawczyk laboratory is funded by R01 EY027011. The authors would also like to acknowledge an unrestricted RPB grant to the University of California, Irvine, Department of Ophthalmology. Work in Ju laboratory is supported by R01 EY031697. The International Centre for Translational Eye Research (MAB/2019/12) project is carried out within the International Research Agendas program of the Foundation for Polish Science co‐financed by the European Union under the European Regional Development Fund.

## CONFLICT OF INTEREST

None.

## Supporting information


FigureS1‐S6

## Data Availability

All sequencing data will be available at GEO database (GSE212189) upon publication of the manuscript.
