# Supplementary material for: Stress induced aging in mouse eye
Source: Aging Cell. 2022 Nov 17;21(12):e13737. doi: 10.1111/acel.13737 (PMC9741506; doi:10.1111/acel.13737)

SUPPLEMENTARY METERIAL FOR:

Stress induced aging in mouse eye

Qianlan Xu[1], Cezary Rydz[1*], Viet Anh Nguyen Huu[2], Lorena Rocha[2], Claudia Palomino La Torre[2], Irene Lee[1], William Cho[1], Mary Jabari[2], John Donello[1], David C. Lyon[3], Bobby Brooke[4], Steve Horvath[4], Robert N. Weinreb[2], Won-Kyu Ju[2], Andrzej Foik[3,5], Dorota Skowronska-Krawczyk[6+]*

[1]Department of Physiology and Biophysics, Center for Translational Vision Research, School of Medicine, University of California, Irvine, Irvine, CA.
[2]Viterbi Family Department of Ophthalmology, Hamilton Glaucoma Center and Shiley Eye Institute, School of Medicine, University of California, San Diego, La Jolla, CA.
[3]Department of Anatomy and Neurobiology, School of Medicine, University of California, Irvine, Irvine, CA, USA.
[4]Epigenetic Clock Development Foundation, Torrance, USA
[5]International Centre for Translational Eye Research, Institute of Physical Chemistry, Polish Academy of Sciences, Warsaw, Poland.
[6]Department of Physiology and Biophysics, Department of Ophthalmology, Center for Translational Vision Research, School of Medicine, University of California, Irvine, Irvine, CA.

Keywords: stress response; aging; retinal ganglion cells; IOP; senescence

Running title: Aging and age-related stress response.

[*] equally contributed

[+]Corresponding author
Dorota Skowronska-Krawczyk, PhD
Department of Physiology and Biophysics
Department of Ophthalmology
Center for Translational Vision Research
University of California Irvine, Gavin Herbert Eye Institute
837 Health Sciences Rd
Irvine, CA 92617
Email: dorotask@hs.uci.edu

Supplementary Figure 1. A. Summary of OMRs at various pattern contrasts. qOMR in scotopic conditions at 75% pattern contrast (left) 3-month-old mice (mean, 1.236 ± 0.078 [SD]; *n* = 8 animals) and 18-month-old mice (mean, 1.134 ± 0.08; *n*=10 animals) (t=2.73, p=0.0076**)**. qOMR in photopic conditions at 50% pattern contrast. 3-month-old mice (mean, 1.35 ± 0.16 [SD]; *n* = 9 animals) and 18-month-old mice (mean, 1.13 ± 0.077; *n*=4 eyes) (t=3.58, p=0.0047**)**, (Welch's unpaired, two-tailed t-test, Mean and SEM are shown. **P < 0.01). **B.** Metascape networks of enriched pathway from up- and down-regulated DEGs during aging. Each node in the figure represents one enriched ontology term. Node size indicates the number of genes in each term. Node color represent its cluster identity and nodes of the same color belong to the same cluster. One term from each cluster is selected to have its term description shown as label in the figure. All the enriched terms were further bounded and annotated as more general classes. **C.** Profile plots and heatmap plots show up- and down-regulation during aging (3-month versus 18-month) in chromatin accessibility (deepTools normalized RPKM with bin size 5) with 2.5Kb flanking distance to center of each region. n = 6 biological replicates. **D.** Motif Analyses performed by using Homer tool identified potential regulators in response to mild IOP in 18-month-old mouse retina from gained accessible regions. The differential analysis was performed by MACS2 with cutoff of q-value at 0.05 and with log2foldchange > 1. n = 6 biological replicates. Top-ranked enriched Homer Known motifs in p-value are listed in the table.

Supplementary Figure 2. A. and C. Representative micrographs from Brn3a-stained retinal flat mounts, RGC count 7 days post-IOP elevation (A) and 21 days post-IOP elevation (C). Scale bar = 100 μm. **B and D.** Quantification of RGC count from retinal micrographs in IOP-treated and non-treated retinas in 3-month, old C57BL/6J mice 7 days post-IOP elevation (D) and 21 days post-IOP elevation. Eyes were exposed to 30, 50, or 90 mmHg pressure for 1h. 7 days post-IOP cohort was exposed to 7 and 15mmHg pressure for 1h. Each data point is an average of 6 independent frames in the central and peripheral retina, represented as % RGC count as compared to contralateral healthy eye (one-way ANOVA, multiple comparisons* P < 0.05, *** P< 0.001).). Dots indicate number of repeats for each stress level. Visual Evoked Potential measurements. **E.** Scheme of the VEP reading, with the measurement electrode in the striate cortex, and the reference in the mouth; measurements were taken unilaterally, with contralateral eye covered with an eye patch. **F.** Quantification of the P1-N1 visual potential from the VEP traces 7 days post-procedure in response to exposure to 7, 15, 30, and 90 mmHg in 3-month-old animals. (one-way ANOVA, multiple comparisons. Mean and SD are shown. *P < 0.05; ***P < 0.001.) **G.** Quantification of the P1-N1 visual potential from the VEP after 21 days. (one-way ANOVA, multiple comparisons. Mean and SD are shown. *P < 0.05; ***P < 0.001). Dots indicate number of mice used for each intraocular pressure level. **H.** Relative change in the VEP N1 latency in response to different levels of hypertension in 3-month-old animals. (one-way ANOVA, multiple comparisons. Mean and SD are shown. *P < 0.05; ***P < 0.001) . **I.** Relative change in the VEP P1 latency in response to different levels of hypertension in 3-month-old animal. Mean and SEM are shown. **I.** *In situ* RNA hybridization staining by RNAscope in focused cell layers of retina in 18-month-old mice 2 days after mild IOP elevation. **J.** ONH images. Dashed line indicates area used for neurofilament count. Statistical significance obtained with t-test. Bar represents mean. ****P < 0.0001. **K.** GSEA enrichment plot of hallmark apoptosis genes and row Z-score heatmap of hallmark apoptosis, senescence, and ECM homeostasis genes in 3-month

and 18-month retinae upon IOP stress versus contralateral control. **L.** Histogram plot shows genes expression foldchange of overlapped DEGs upon IOP in both 3-month and 18-month-old mouse retina.

Supplementary Figure 3. RNAscope analysis of expression of *p16Ink4a* and *p19Arf* in retinal neurons in old non-treated control and IOP-treated retina 2 days after IOP treatment and HiPlex analysis of expression of set of genes from inflammaging and senescence pathways in old control non-treated retinas and IOP-treated retinas. Size bar 20μm.

Supplementary Figure 4. A. Profile plots and heatmap plots for chromatin accessibility intensities (deepTools normalized RPKM with bin size 5) over all up-regulated enhancer regions (2.5Kb flanking distance to center of each region). **B.** Motif analyses performed by using Homer tool identified potential regulators in response to mild IOP in 18-month-old mouse retina from gained accessible regions. The differential analysis was performed by MACS2 with cutoff of q-value at 0.05 and with log2foldchange > 1. n = 3 biological replicates. Top-ranked enriched Homer Known motifs in p-value are listed in the table. **C.** R-square plots illustrate the correlation between transcription activation and chromatin accessibility changes upon mild IOP treatment in 18-month-old mouse retinae on Activity-by-contact (ABC) index of both promoters and predicted enhancers with weighted calculation by known *cis*-looping contacts for selected pathways.

Supplementary Figure 5. A. The bar plot showed normalized enrichment scores (NES) of all defined significant enriched (nominal p-value <5%) hallmark gene sets from pre-ranked GSEA in 3-month-old wildtype mouse retinae upon multiple IOP treatments. n = 2 biological replicates. NES determines the magnitude of enrichment of each hallmark gene set across all analyzed gene sets. The statistical significance in nominal (NOM) p-value were indicated by a discrete color scale**. B.** Metascape networks of enriched pathway from up-regulated DEGs in 3-month-old mouse retinae upon multiple IOP treatments. Each node in the figure represents one enriched ontology term. Node size indicates the number of genes in each term. Node color represent its cluster identity and nodes of the same color belong to the same cluster. One term from each cluster is selected to have its term description shown as label in the figure. **C.** Heatmap of row z-score of RPKM of each gene in gene set Apoptosis with its pre-ranked GSEA enrichment plot on top the heatmap and curated senescence pathway genes (right).

Supplementary Figure 6. Box plots showing the expression of selected genes which levels of regulatory elements DNA methylation was reversely correlated with gene transcription. N=3, p-values presented on the figure. RPKM - Reads Per Kilobase of transcript, per Million mapped reads.

Supplementary Figure 1

A

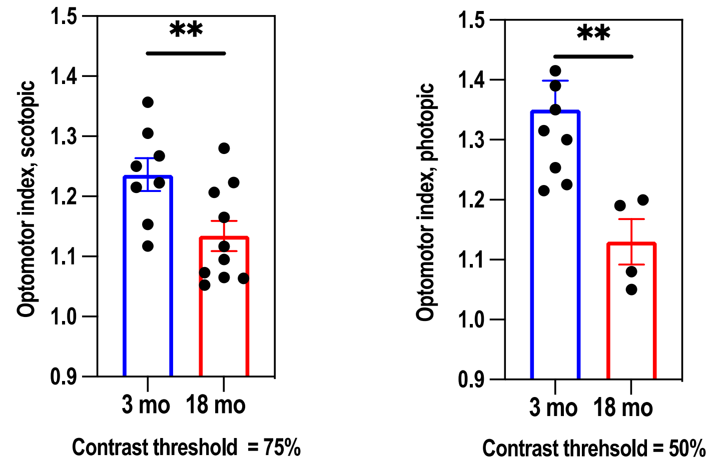

B

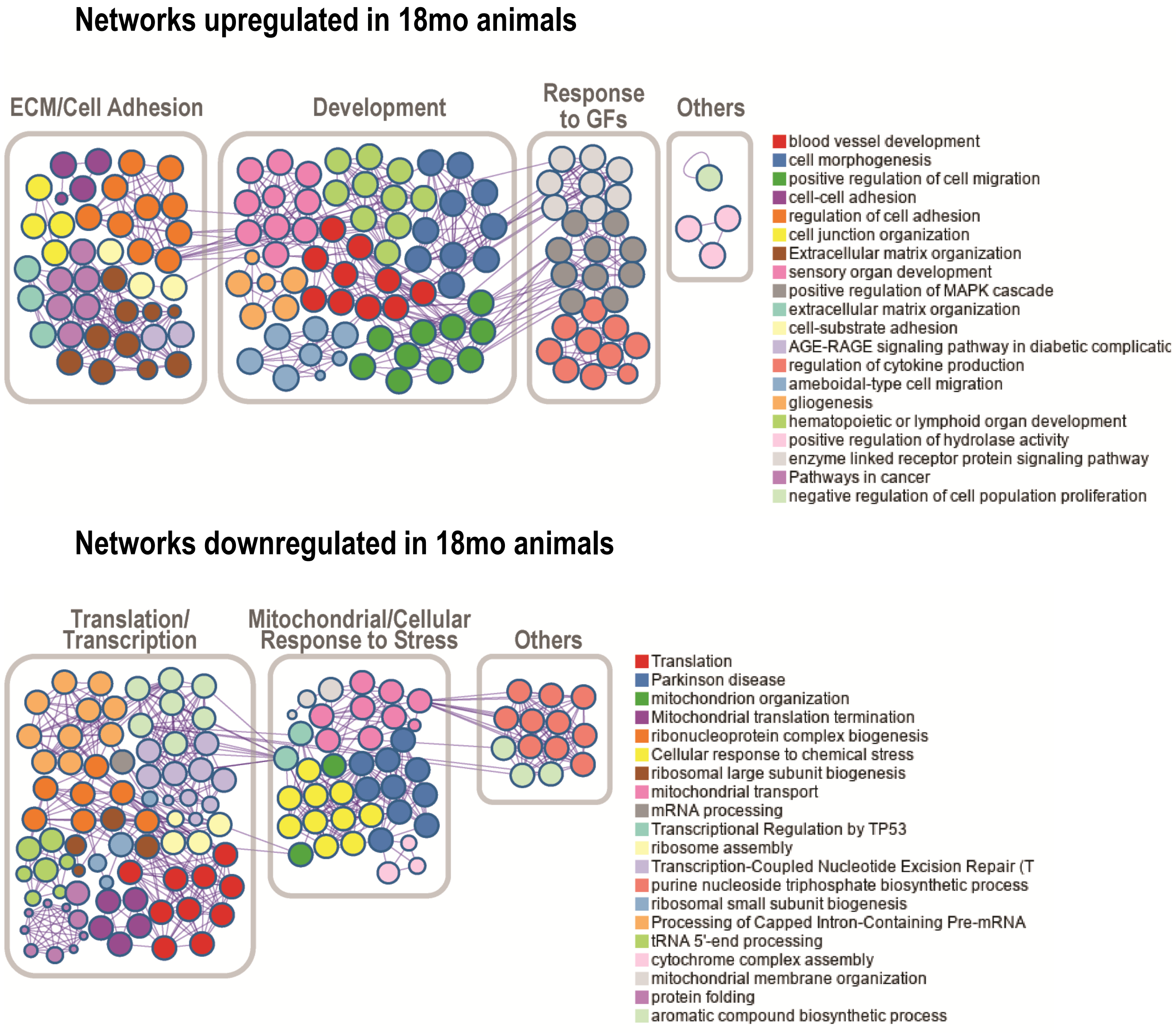

C

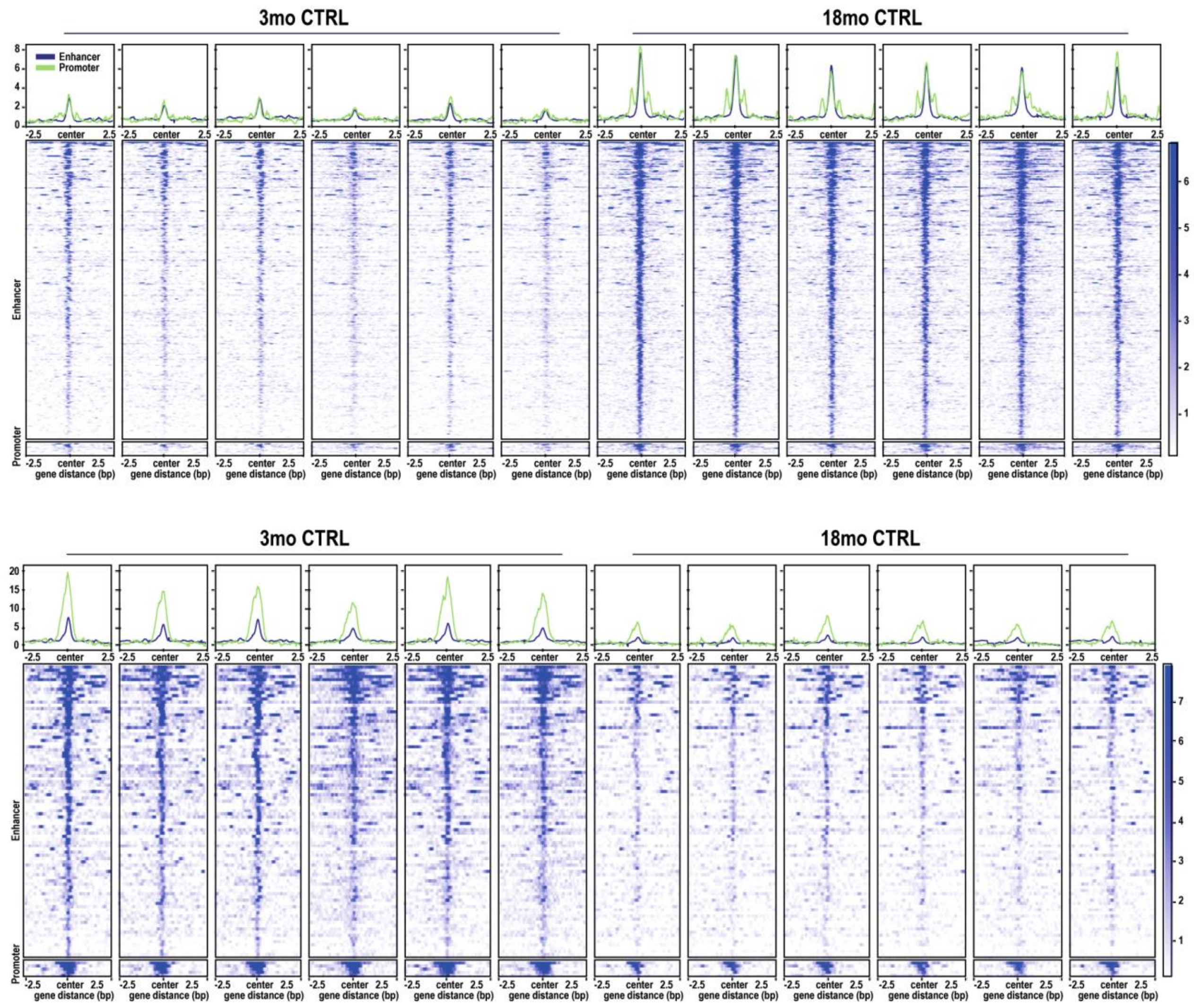

D

Motif Analyses identified potential regulators during retina aging

Up-regulated peaks Enriched Homer Known Motifs (top-ranked)

Rank	Motif	Name	P-value
1	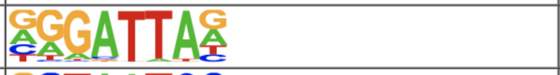	GSC(Homeobox)/FrogEmbryos-GSC-ChIP-Seq(DRA000576)/Homer	1e-156
2	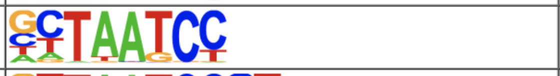	CRX(Homeobox)/Retina-Crx-ChIP-Seq(GSE20012)/Homer	1e-125
3	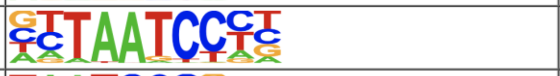	Otx2(Homeobox)/EpiLC-Otx2-ChIP-Seq(GSE56098)/Homer	1e-114
4	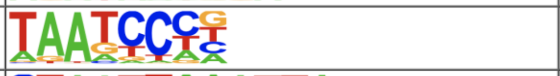	Pitx1(Homeobox)/Chicken-Pitx1-ChIP-Seq(GSE38910)/Homer	1e-49
5	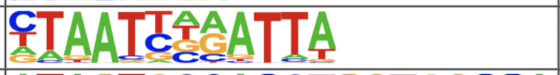	Phox2a(Homeobox)/Neuron-Phox2a-ChIP-Seq(GSE31456)/Homer	1e-25
6	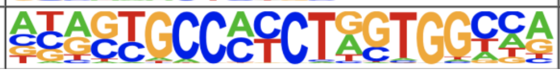	CTCF(Zf)/CD4+-CTCF-ChIP-Seq(Barski_et_al.)/Homer	1e-25

Down-regulated peaks Enriched Homer Known Motifs (top-ranked)

Rank	Motif	Name	P-value
1	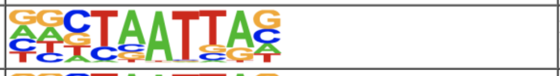	En1(Homeobox)/SUM149-EN1-ChIP-Seq(GSE120957)/Homer	1e-39
2	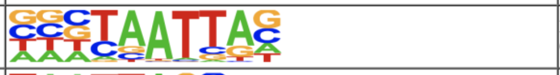	DLX2(Homeobox)/BasalGanglia-Dlx2-ChIP-seq(GSE124936)/Homer	1e-39
3	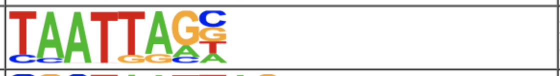	Lhx2(Homeobox)/HFSC-Lhx2-ChIP-Seq(GSE48068)/Homer	1e-38
4	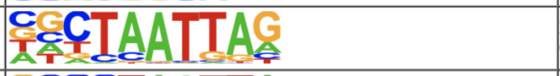	LHX9(Homeobox)/Hct116-LHX9.V5-ChIP-Seq(GSE116822)/Homer	1e-35
5	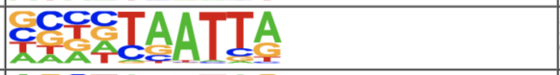	DLX1(Homeobox)/BasalGanglia-Dlx1-ChIP-seq(GSE124936)/Homer	1e-34
6	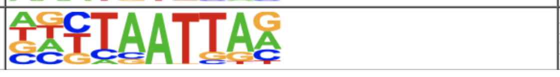	Lhx1(Homeobox)/EmbryoCarcinoma-Lhx1-ChIP-Seq(GSE70957)/Homer	1e-33

Supplementary Figure 2

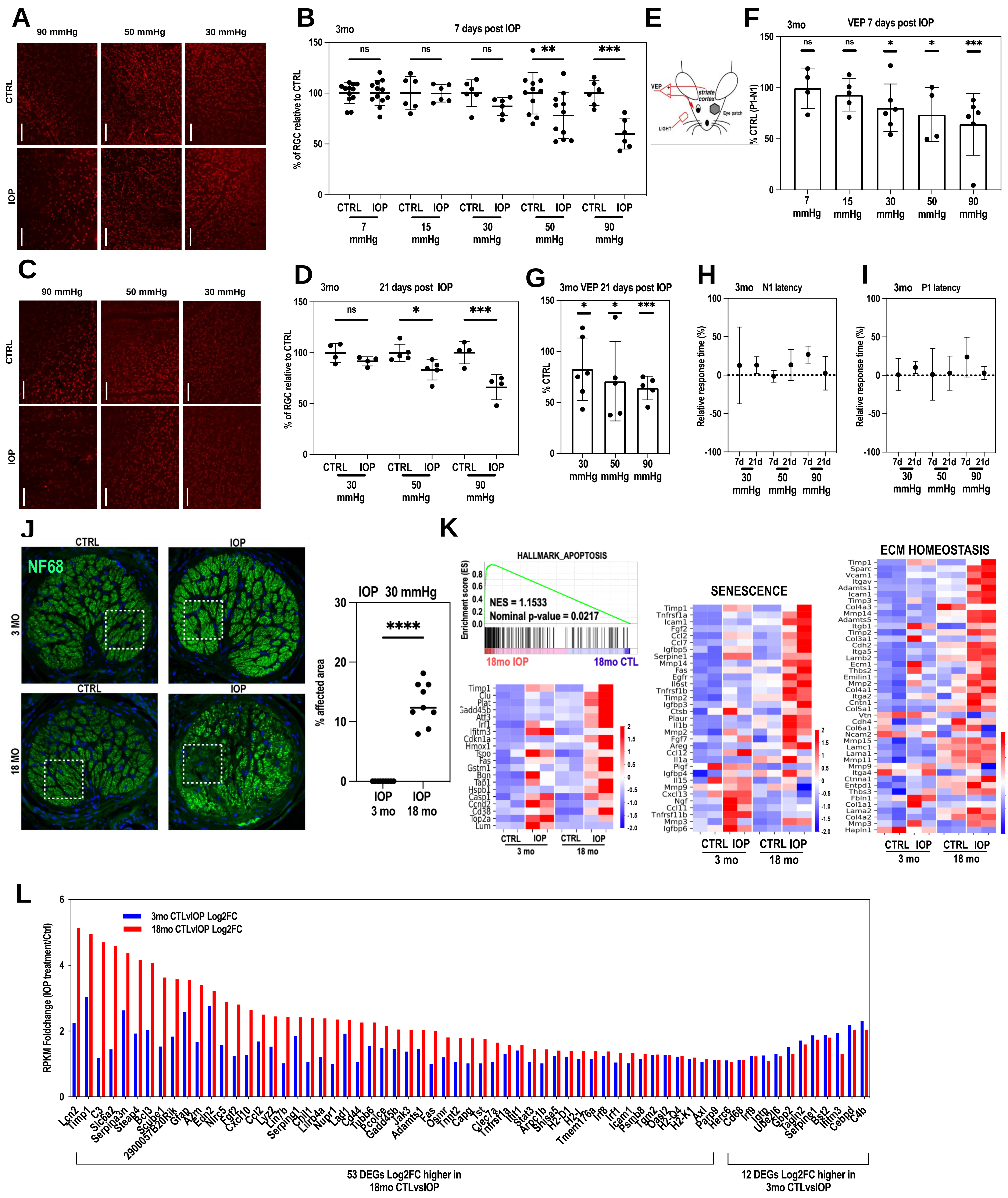

Supplementary Figure 3

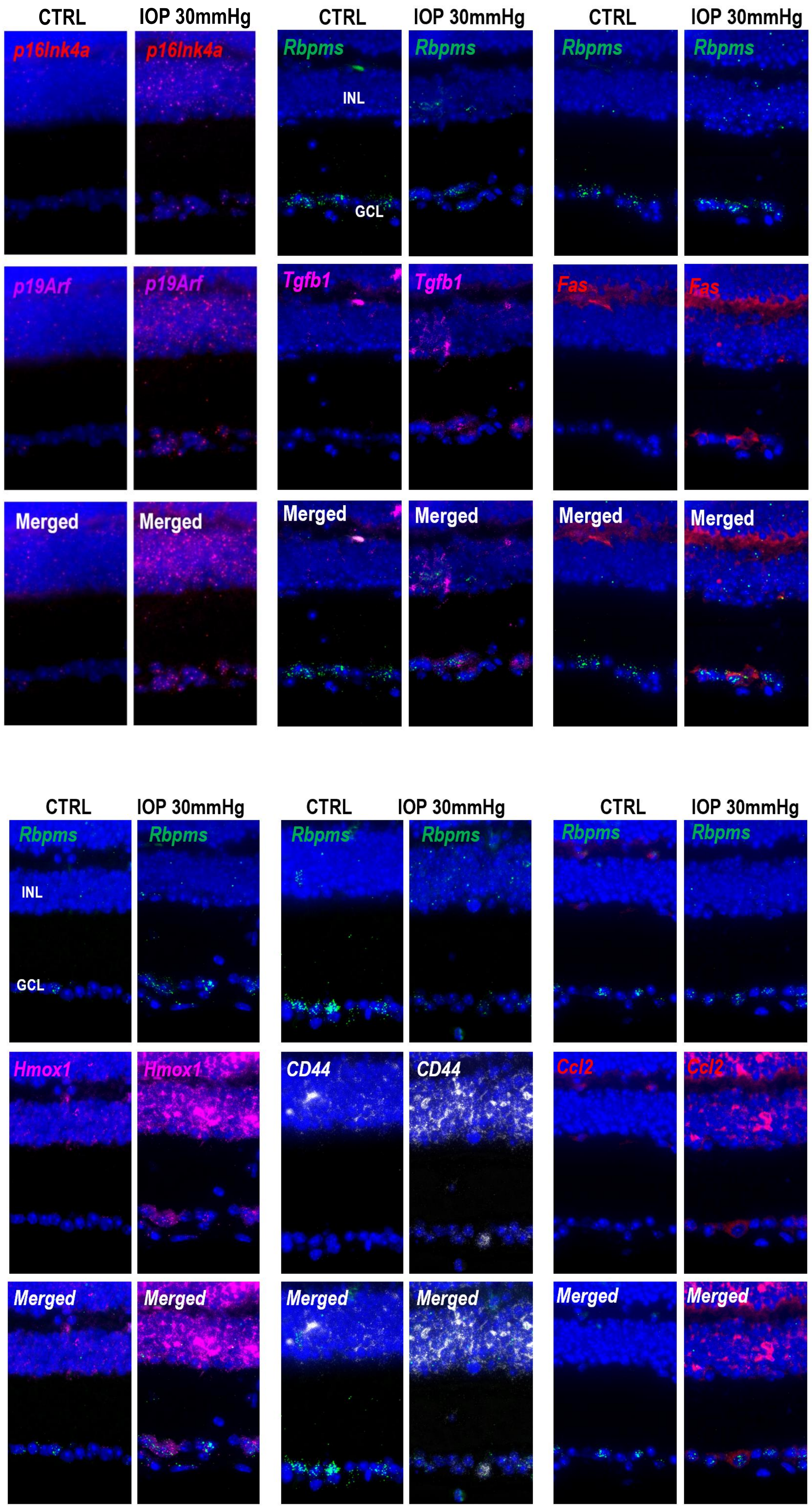

Supplementary figure 4

A

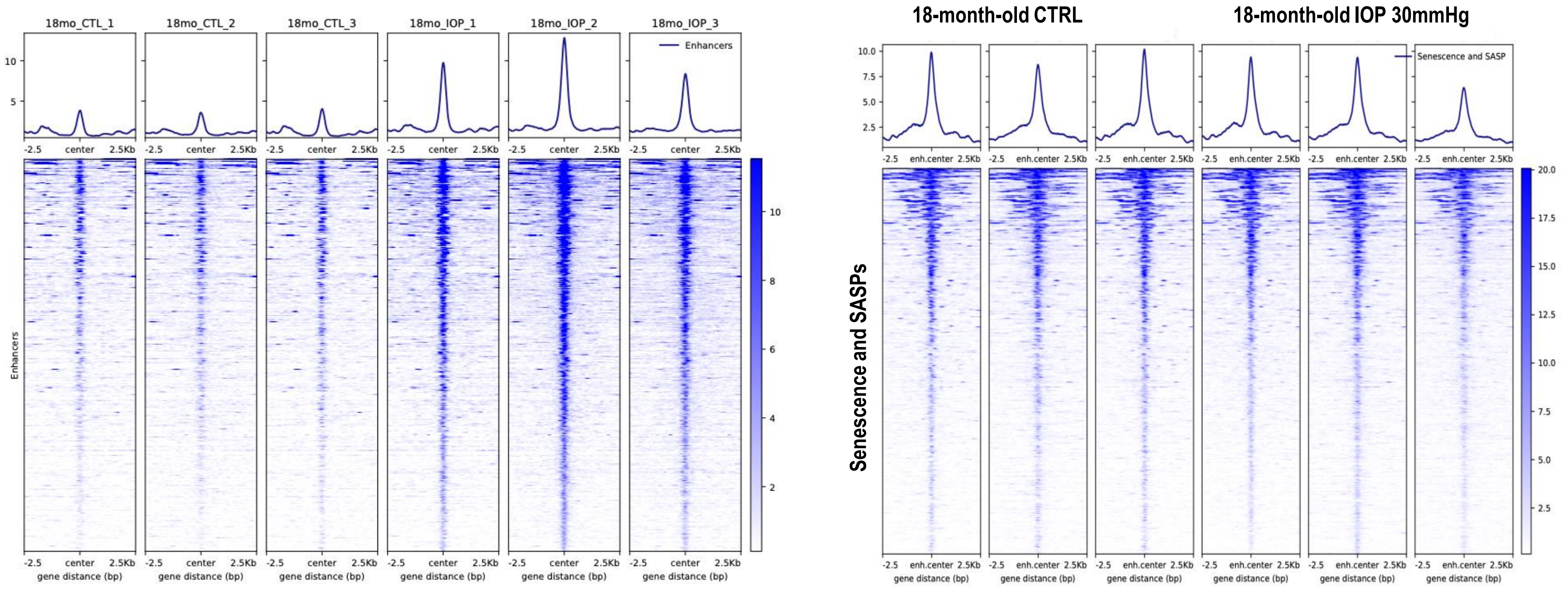

B

Motif Analyses identified potential regulators upon IOP in 18mo

<u>18mo CTL (N=3) vs 18mo IOP (N=3)</u> (MACS2, edgeR, FDR q-value<0.05, $Log_2FoldChange > 1$)

Up-regulated peaks Enriched <u>Homer Known</u> Motifs (top-ranked)

Rank	Motif	Name	P-value	log P-pvalue	q-value (Benjamini)	# Target Sequences with Motif	% of Targets Sequences with Motif	# Background Sequences with Motif	% of Background Sequences with Motif
1	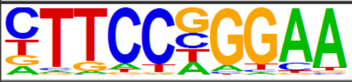	Stat3(Stat)/mES-Stat3-ChIP-Seq(GSE11431)/Homer	1e-51	-1.194e+02	0.0000	90.0	35.16%	2295.9	4.74%
2	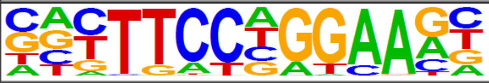	Stat3+il21(Stat)/CD4-Stat3-ChIP-Seq(GSE19198)/Homer	1e-49	-1.137e+02	0.0000	102.0	39.84%	3371.2	6.96%
3	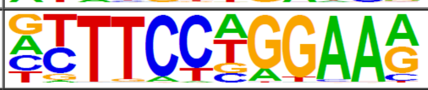	STAT4(Stat)/CD4-Stat4-ChIP-Seq(GSE22104)/Homer	1e-44	-1.036e+02	0.0000	107.0	41.80%	4223.1	8.71%
4	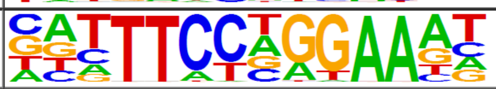	STAT1(Stat)/HelaS3-STAT1-ChIP-Seq(GSE12782)/Homer	1e-40	-9.321e+01	0.0000	60.0	23.44%	1136.2	2.34%
5	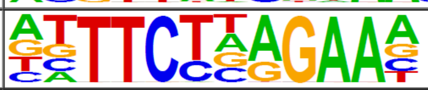	STAT5(Stat)/mCD4+-Stat5-ChIP-Seq(GSE12346)/Homer	1e-28	-6.611e+01	0.0000	53.0	20.70%	1395.0	2.88%
6	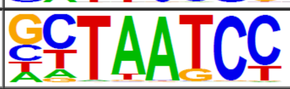	CRX(Homeobox)/Retina-Crx-ChIP-Seq(GSE20012)/Homer	1e-25	-5.976e+01	0.0000	155.0	60.55%	13772.5	28.42%
7	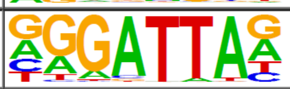	GSC(Homeobox)/FrogEmbryos-GSC-ChIP-Seq(DRA000576)/Homer	1e-24	-5.747e+01	0.0000	108.0	42.19%	7259.9	14.98%
8	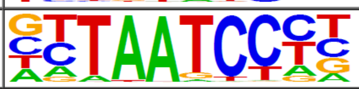	Otx2(Homeobox)/EpiLC-Otx2-ChIP-Seq(GSE56098)/Homer	1e-24	-5.747e+01	0.0000	89.0	34.77%	5015.5	10.35%
9	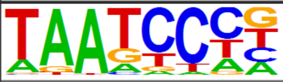	Pitx1(Homeobox)/Chicken-Pitx1-ChIP-Seq(GSE38910)/Homer	1e-17	-4.128e+01	0.0000	191.0	74.61%	23062.1	47.59%
10	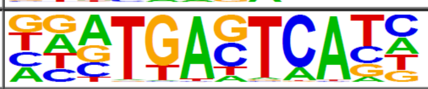	Fra1(bZIP)/BT549-Fra1-ChIP-Seq(GSE46166)/Homer	1e-17	-4.050e+01	0.0000	52.0	20.31%	2371.5	4.89%

C

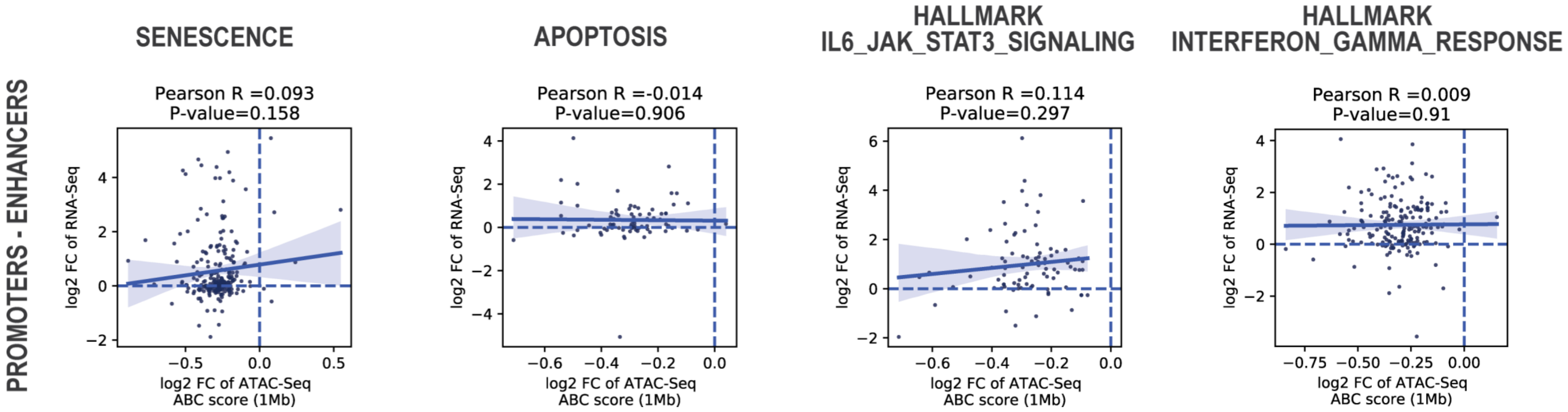

Supplementary figure 5

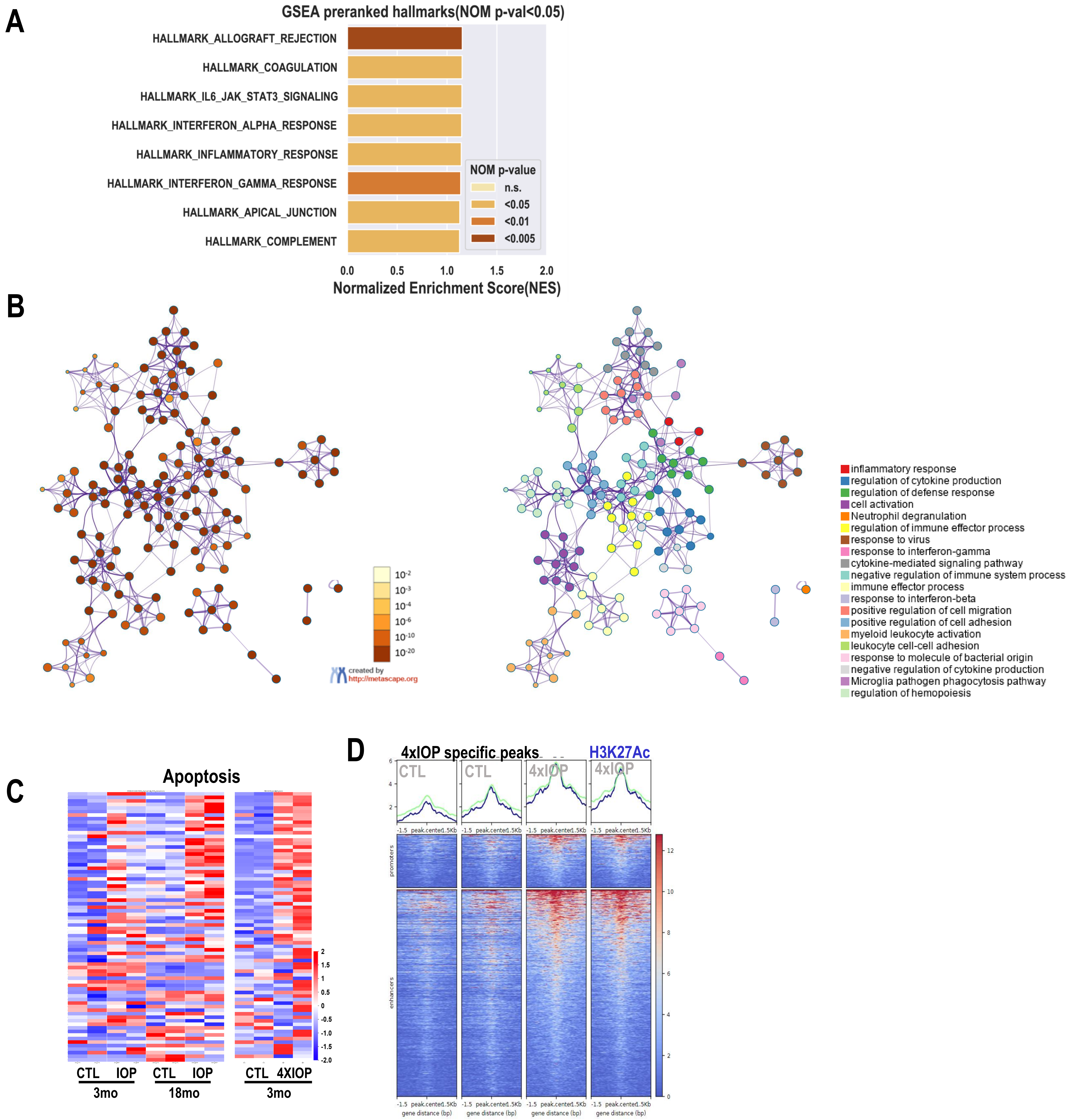

Supplementary Figure 6

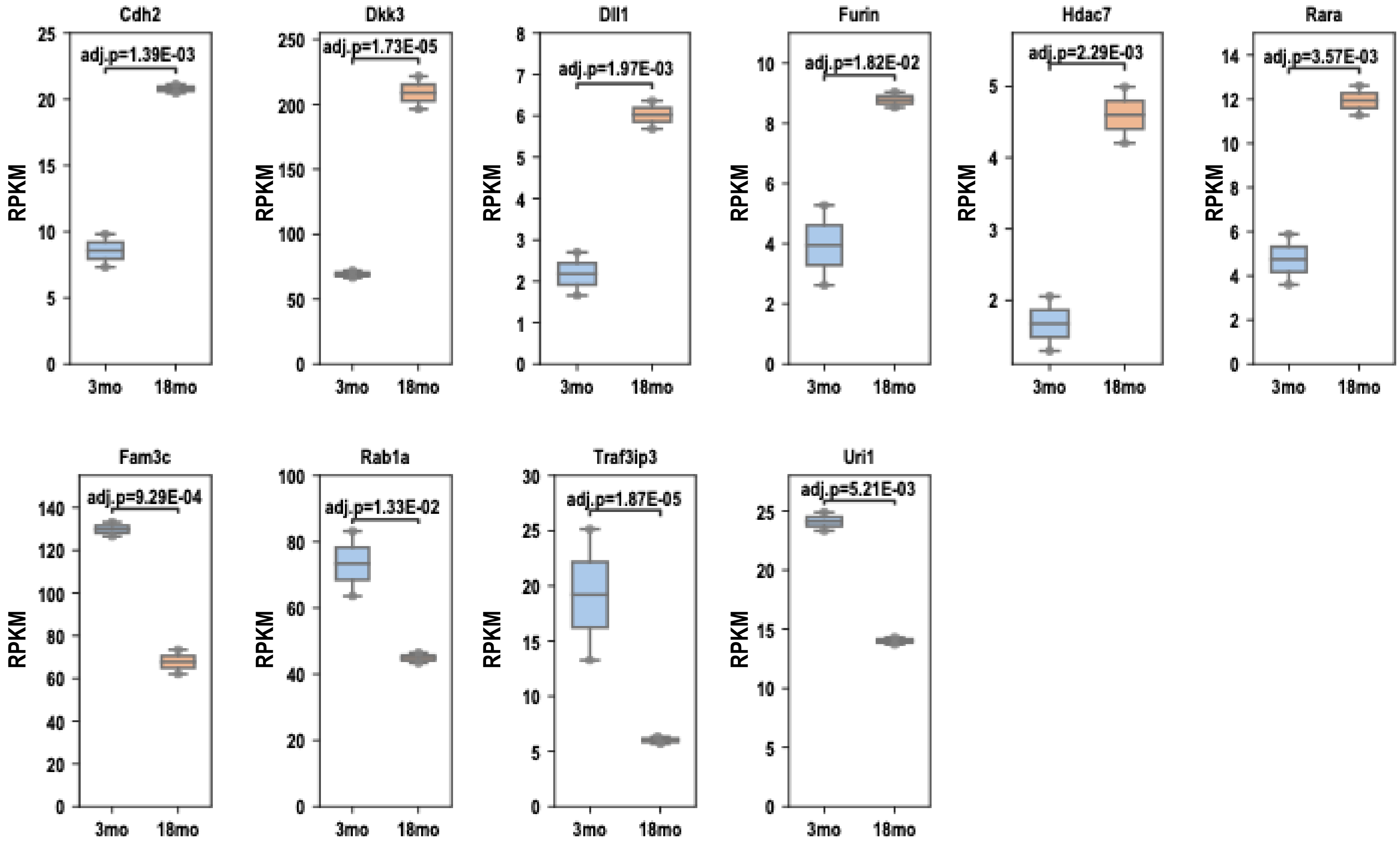

Supplement: Supplementary file 1 — FigureS1‐S6 [file ACEL-21-e13737-s001.pdf]
